# Cytofkit: A Bioconductor Package for an Integrated Mass Cytometry Data Analysis Pipeline

**DOI:** 10.1371/journal.pcbi.1005112

**Published:** 2016-09-23

**Authors:** Hao Chen, Mai Chan Lau, Michael Thomas Wong, Evan W. Newell, Michael Poidinger, Jinmiao Chen

**Affiliations:** Agency for Science, Technology and Research (A*STAR), Singapore Immunology Network (SIgN), Singapore; Hebrew University of Jerusalem, ISRAEL

## Abstract

Single-cell mass cytometry significantly increases the dimensionality of cytometry analysis as compared to fluorescence flow cytometry, providing unprecedented resolution of cellular diversity in tissues. However, analysis and interpretation of these high-dimensional data poses a significant technical challenge. Here, we present cytofkit, a new Bioconductor package, which integrates both state-of-the-art bioinformatics methods and in-house novel algorithms to offer a comprehensive toolset for mass cytometry data analysis. Cytofkit provides functions for data pre-processing, data visualization through linear or non-linear dimensionality reduction, automatic identification of cell subsets, and inference of the relatedness between cell subsets. This pipeline also provides a graphical user interface (GUI) for ease of use, as well as a shiny application (APP) for interactive visualization of cell subpopulations and progression profiles of key markers. Applied to a CD14^−^CD19^−^ PBMCs dataset, cytofkit accurately identified different subsets of lymphocytes; applied to a human CD4^+^ T cell dataset, cytofkit uncovered multiple subtypes of T_FH_ cells spanning blood and tonsils. Cytofkit is implemented in R, licensed under the Artistic license 2.0, and freely available from the Bioconductor website, https://bioconductor.org/packages/cytofkit/. Cytofkit is also applicable for flow cytometry data analysis.

This is a *PLOS Computational Biology Software* Article.

## Introduction

Mass cytometry, or cytometry by time-of-flight (CyTOF), uniquely combines metal-labeling of antibodies with mass spectrometry to enable high-dimensional measurement of the characteristics of individual cells [1,2]. The high purity and choice of metal isotopes overcome the limitations of spectral overlap in flow cytometry, and allow for simultaneous analysis of more than 40 markers per cell [3,4]. This technology has been successfully applied in a number of areas including mapping phenotypic heterogeneity of leukemia [5], inferring cellular progression and hierarchies [6], assessing drug effects on immune cells [7,8] and uncovering mechanisms of cellular reprogramming [9]. Despite the advantages of mass cytometry, effective analysis and interpretation of these high dimensional and large-scale datasets remain challenging. Traditional manual gating, the gold-standard method for flow cytometry data analysis, is not practical for mass cytometry due to its high dimensionality. In addition, most automated methods designed for flow cytometry data do not perform well for mass cytometry data [10].

Analysis of mass cytometry data has several key challenges including debarcoding [11], batch normalization [12], visualization of high-dimensional data, identification of cell subsets, inference of relatedness between cell subsets, and detection of changes in subset abundance. This manuscript focuses on addressing the following three key challenges for data that don’t display batch effect. The first challenge is efficient visualization of these high-dimensional data. A biaxial plot that displays the correlation of every two markers is a common way to visualize flow cytometry data. With the fact that *m*(*m* − 1)/2 biaxial plots are needed to fully visualize an m-dimensional dataset, this approach is impractical for mass cytometry data as the parameter m of mass cytometry is usually greater than 40. Alternative dimensionality reduction approaches have been used to transform the high-dimensional data to a low-dimensional representation, thus allowing visualization of the cells in a single plot. In Newell *et al*. [13], principal component analysis (PCA) was used to visualize a 25-parameter mass cytometry panel for CD8^+^ T cells. However, PCA is a linear transformation, and it cannot capture nonlinear relationships. To address this limitation, Amir *et al*. [5] developed a visualization tool named viSNE which utilizes the t-distributed stochastic neighbor embedding (t-SNE) algorithm. t-SNE is a nonlinear dimensionality reduction approach [14] which embeds the data from high dimensional space into a lower dimensional map based on similarities. On a t-SNE map, similar cells are placed to nearby points, while dissimilar cells are placed far apart. It has been demonstrated that t-SNE can effectively visualize cellular heterogeneity in normal and leukemic bone marrow [5].

The second challenge is to identify cell subpopulations. To address this challenge, the ACCENSE method has been developed to automatically identify cellular subpopulations using a density peak-finding algorithm on a t-SNE transformed 2-D map [15]. However, not all cells are assigned to a defined subpopulation in this method. DensVM extends ACCENSE by using support vector machine (SVM) to assign any unassigned cells to the subpopulations in a machine learning manner [16]. This approach has been demonstrated to precisely detect the boundaries of cell populations in murine myeloid data. DensVM has also been applied to map the numerous subtypes of follicular helper T cells derived from human blood and tonsils [16,17]. However, both ACCENSE and DensVM rely on a computationally intensive search for an optimal number of subpopulations. PhenoGraph, a graph-based partitioning method, has demonstrated efficiency in subpopulation detection [10]. PhenoGraph first constructs a nearest-neighbor graph which captures the phenotypic relatedness of the high-dimensional data, and then it applies a graph partition algorithm called Louvain [18] to dissect the nearest-neighbor graph into phenotypically coherent subpopulations. Applied to the study of acute myeloid leukemia, PhenoGraph provided a comprehensive view of the major phenotypes and elucidated intra- and inter-tumor heterogeneity. PhenoGraph has also been tested on three different mass cytometry datasets of healthy human bone marrow, and it displayed superior accuracy and robustness in immune cell type detection as compared to other methods.

The third challenge is to detect cellular progression. In addition to defining distinctive cell subsets, there is great interest in resolving the order of cellular differentiation to reveal their developmental relationships. For example, Bendall *et al*. developed a graph-based trajectory detection algorithm named Wanderlust, which orders cells into a unified trajectory that reflects the developmental path [19]. This method correctly predicted the early developmental path of human B-lymphocytes. Nevertheless, this algorithm was designed for linear and non-branching developmental path, and hence is less useful for interpreting complex single-cell data with multiple developmental lineages. Wishbone extended the ability of Wanderlust to capture bifurcating developmental trajectories through introducing waypoints and identifying branch points [20]. Wishbone is based on diffusion map, which has been demonstrated to be powerful and robust for detecting the global geometric structures from the data [21]. However, Wishbone requires the input of a starting cell. SPADE is an innovative approach designed to extract cellular hierarchy using minimum spanning tree (MST) [6]. While SPADE enables the prediction of multi-branched cell developmental pathways, the hierarchical clustering used in SPADE needs a pre-specification of the number of clusters, additionally, the MST used by SPADE is susceptible to over-fitting and is not robust for local variation [9]. Our recent novel method named Mpath constructs multi-branching cell lineages from single-cell data using neighborhood-based cell state transitions [22]. However we have only demonstrated its applications for single-cell RNA-sequencing data. We are currently testing and optimizing Mpath for mass cytometry and flow cytometry data.

In this report, we present an integrated analysis pipeline, named cytofkit. It is designed to analyze mass cytometry data in four main steps. In the first step, cytofkit performs data pre-processing, and enables combined analysis of multiple Flow Cytometry Standard (FCS) files. Users are allowed to customize their data merging strategy to combine the data using selectable transformation methods. The remaining three steps address respectively, each of the three challenges discussed above. Firstly, cytofkit provides state-of-the-art clustering methods including DensVM [16], FlowSOM [23] and PhenoGraph [10], as well as an in-house newly developed algorithm named ClusterX for automatic detection of cell subpopulations. Secondly, it provides functions to visualize the high-dimensional data with color-labeled cell types using either linear transformation such as PCA or non-linear dimensionality reduction such as ISOMAP [24], diffusion map or t-SNE (we use Barnes-Hut variant of t-SNE, a speed optimized implementation of t-SNE [25]). Lastly, it infers the relatedness between cell subsets using ISOMAP or diffusion map. In addition to providing an integrated analysis pipeline, cytofkit provides a user-friendly GUI and an interactive shiny APP to facilitate result exploration and interpretation. Through the application of cytofkit to a CD14^−^CD19^−^ PBMCs dataset, cytofkit was able to accurately identify known populations of lymphocytes including CD4^+^, CD8^+^, γδT, NK, and NKT cells, and further segregate these subsets to reveal subpopulations such as different stages of CD4^+^ and CD8^+^ T cell differentiation, as well as three subsets of γδT and two subsets of NK cells. Moreover, as shown in our previous publication [17], application of cytofkit for an objective comparison of human T helper (TH) cells derived from peripheral blood versus tonsils revealed numerous subtypes of follicular helper T cells (T_FH_) cells that followed a continuum spanning both blood and tonsils.

## Design and Implementation

**Fig 1:**
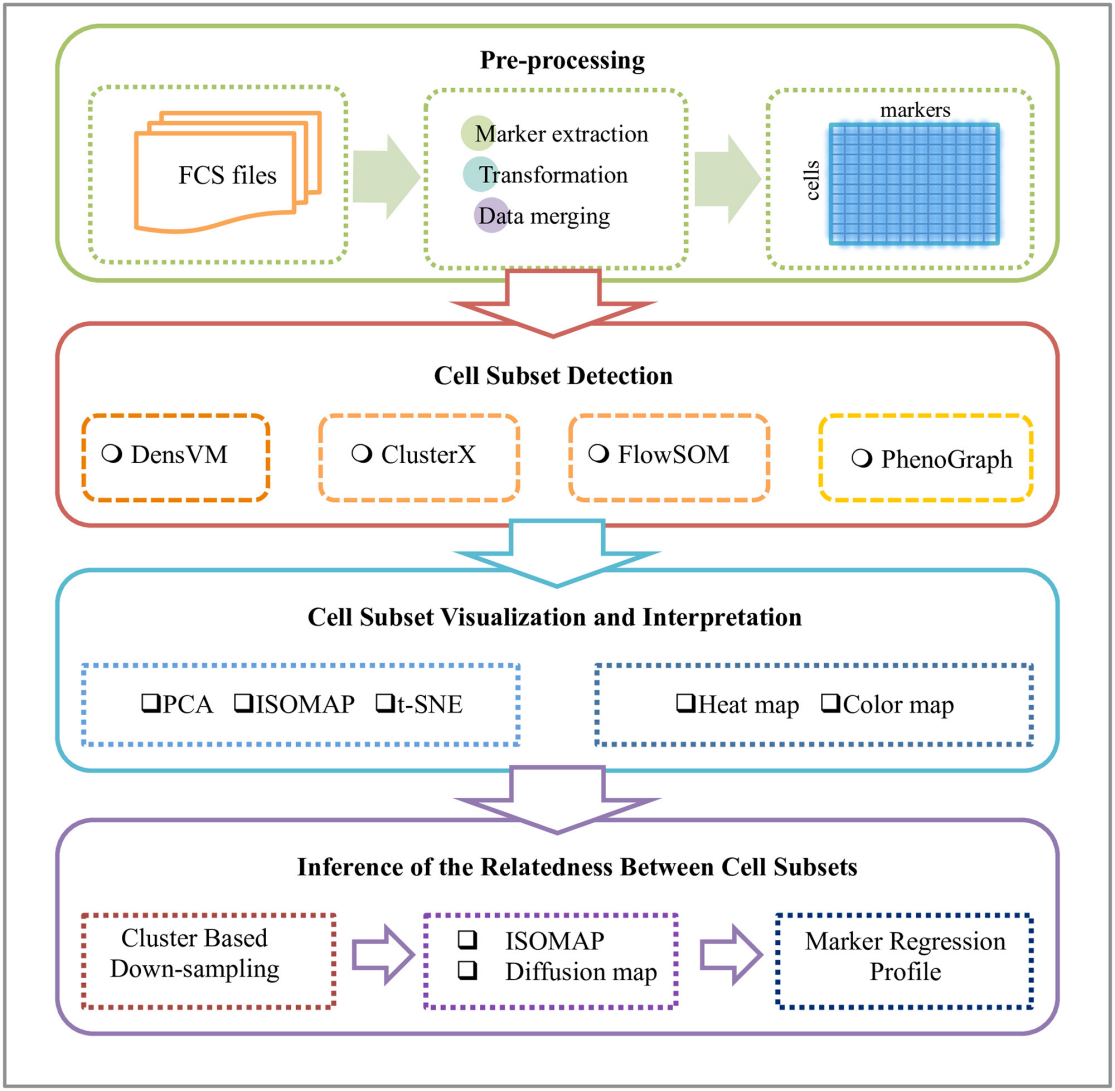
Schematic view of cytofkit pipeline. The cytofkit pipeline consists of four major components: (1) pre-processing, (2) cell subset detection, (3) cell subset visualization and interpretation and (4) inference of the relatedness between cell subsets.

We have developed an integrated mass cytometry data analysis pipeline as an open-source R/Bioconductor package called cytofkit. As shown in Fig 1, the pipeline consists of four major components: (1) pre-processing, (2) cell subset detection, (3) cell subset visualization and interpretation and (4) Inference of the relatedness between cell subsets.

### Pre-processing

Pre-processing is performed on one or multiple FCS files involving three steps to generate the expression matrix. Firstly, expression values of user selected markers are extracted from each FCS file; secondly the extracted data are transformed using either negative value pruned inverse hyperbolic sine transformation (cytofAsinh) or automatic logicle transformation (autoLgcl) [26] (see details in S1 file); finally expression matrixes from each FCS file are combined into a single matrix using one of the four selectable strategies, including i) *ceil* which samples up to a user specified number of cells without replacement from each FCS file, ii) *all* which takes all cells from each FCS file, iii) *min* which samples the minimum number of cells among all the selected FCS files from each FCS file and iv) *fixed* which samples an user specified number of cells (with replacement when the total number of cell in the file is less than the specified number) from each FCS file. In the combined expression matrix, each cell is given a unique ID, which is the concatenation of its original FCS file name and its sequence ID in the file.

### Cell subset detection

The subset detection is implemented by clustering algorithms. Cytofkit provides three state-of-the-art clustering methods DensVM [16], PhenoGraph [10], FlowSOM [23] and one in-house developed clustering algorithm called ClusterX. DensVM and ClusterX are density-based clustering algorithms, which are applied to the t-SNE embedded map, whereas PhenoGraph is a graph based clustering algorithm, which works directly on the high-dimensional data.

#### DensVM

DensVM (Density-based clustering aided by support Vector Machine) is an extension of ACCENSE’s density-based clustering algorithm [15]. ACCENSE’s clustering algorithm first computes 2D probability density from the t-SNE map using the Gaussian kernel transform. A 2D peak-finding algorithm is then applied to identify local density maxima that represent the center of cellular subpopulations. For each peak k, the nearest neighboring peak is identified and distance to the nearest neighbor d_k_ is calculated. ACCENSE then draws a circle of radius d_k_/2 centered at the peak k, and assign all cells within the circle to cluster k. By using this approach, a significant number of cells are located outside any circle and left unclassified, which hampers the estimation of subpopulation frequencies and downstream statistical tests. DensVM overcomes this limitation by utilizing a machine-learning algorithm called support vector machine to train a classifier that learns the patterns of cells that were assigned to ACCENSE clusters. The trained classifier then takes as an input the marker expression profiles of unclassified cells and assigns each of them to one of the ACCENSE clusters based on the assumption that cells from the same cluster should share similar patterns of marker expression (details in paper [16]). DensVM is able to objectively assign every cell to an appropriate cluster.

#### PhenoGraph

PhenoGraph works on an m-by-N intensity matrix, which comprises m parameters of N cells. For each cell, PhenoGraph first identifies k nearest neighbors using Euclidean distance, resulting in N sets of k-neighbors. Based on the number of neighbors shared by every two cells, it calculates the similarity between cells using the Jaccard similarity coefficient and generates a cell-cell similarity matrix, which is then converted into a network. Subsequently, PhenoGraph partitions the network using the Louvain algorithm to extract communities with optimal modularity [18]. This algorithm makes no assumption about the size or number of subpopulations, which make it applicable to many different datasets. In cytofkit, we converted the original python code of PhenoGraph into R script.

#### ClusterX

**Fig 2:**
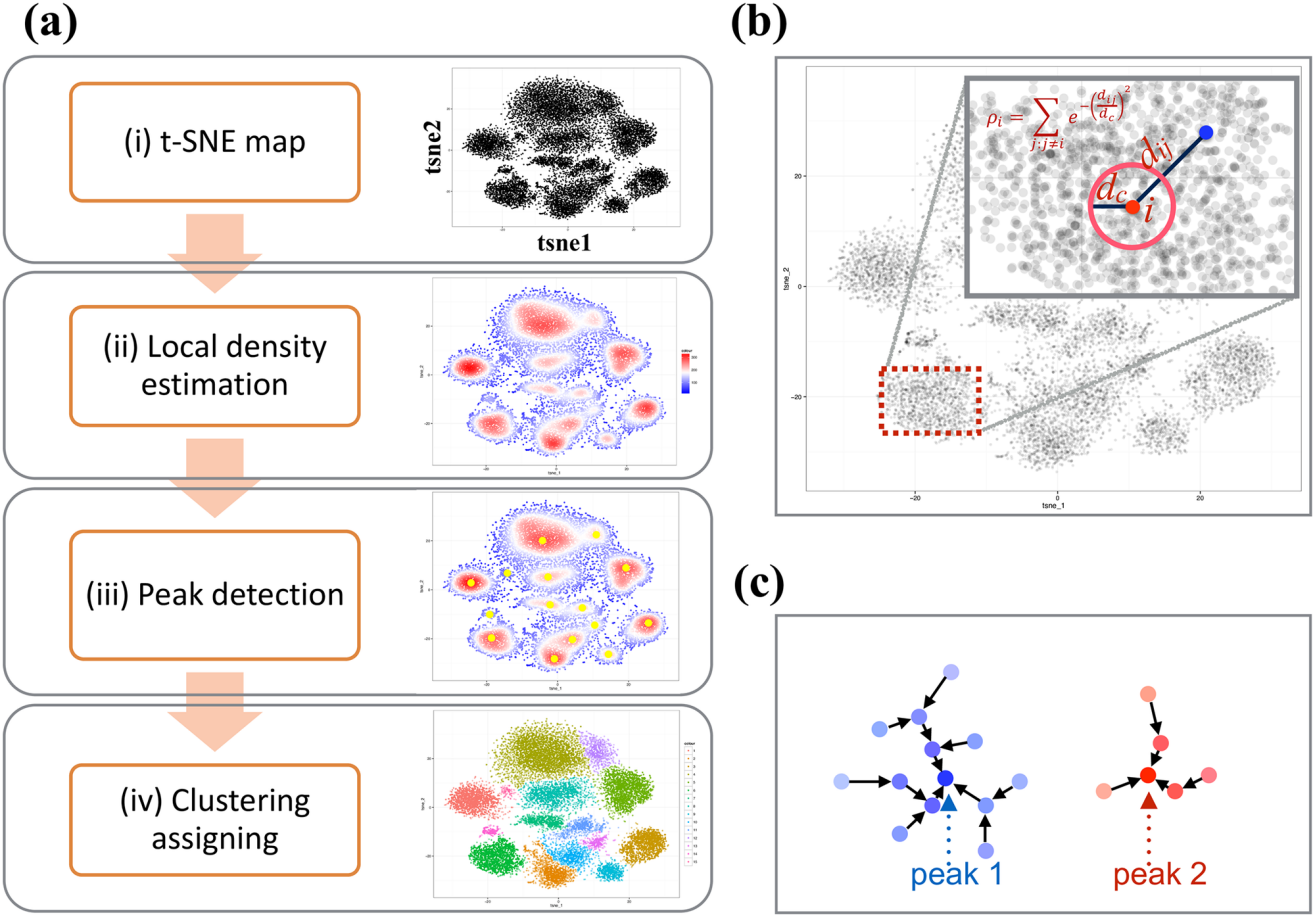
Workflow of ClusterX for mass cytometry data clustering. (a) depict the workflow of ClusterX for mass cytometry data clustering, which contains four steps: (i) t-SNE dimensionality reduction (ii) estimate the local density on the t-SNE map (iii) detect the density peaks represented as cluster centers and (iv) assign the remaining cells to clusters. (b) Explains the local density estimation method. (c) Illustrate the cluster assigning step using two peaks, peak1 and peak 2. Each point is a cell and the color intensity represents the local density of the cell. Then each cell is assigned to be the same cluster as its nearest neighbor cell which has higher density than it.

ClusterX is a clustering method improved from Clustering by fast search and find of density peaks (CFSFDP) [27]. The CFSFDP algorithm is fast and able to recognize clusters regardless of their shape. However it has two main limitations. The first limitation is that it takes a dissimilarity matrix as the input, which results in an O(n^2^) memory burden for a dataset of n cells. The second is that it requires manually decided cut-off values to determine density peaks, which is inefficient and subjective. ClusterX addresses the memory issue with a split-apply-combine strategy [28], and automates density peak detection using generalized (extreme Studentized deviate) ESD test [29]. When combined with t-SNE, ClusterX extends its capacity for clustering high-dimensional data. The workflow of ClusterX for mass cytometry data clustering is illustrated in Fig 2 (see detailed description of ClusterX in S1 file).

### Cell subset visualization and interpretation

Three dimensionality reduction methods are integrated into cytofkit for visualizing the high dimensional mass cytometry data. These include one linear transformation method PCA and two non-linear transformation methods ISOMAP and t-SNE. After dimensionality reduction, cytofkit plots the transformed two-dimensional maps with point color representing the cell type detected from cluster analysis and point shape representing which sample (i.e. FCS file) the cell belongs to. The expression pattern of a specified marker can also be visualized on the dimensionality-reduced map with values represented by colors. A heat map is generated to visualize the median expression level of each marker in each cell type. This heat map facilitates the annotation of known cell types based on prior knowledge of cell type specific marker expression, as well as the detection of novel cell types with novel expression patterns. The percentage of cells in each cluster for each FCS file can also be visualized using a heat map, which helps the detection of changes in abundance of subsets among different samples. All these plots can be either saved automatically by the cytofkit package or interactively visualized with our specifically designed shiny APP (see in **Pipeline Implementation** section). Example t-SNE plots and heat map plots can be found in the **Results and Discussion** section.

### Inference of inter-subset relatedness

Instead of directly estimating cellular developmental path from individual cells, which is computationally challenging and error prone, cytofkit provides assistant approaches for inferring the progression based on the relationship of cell subsets. As we will demonstrate later in our **Results and Discussion** section, ISOMAP or diffusion map perform better for reserving the global inter-relatedness between cell subsets compared to tSNE. ISOMAP takes into account local distances between similar cells and is able to capture the global geometry between different cell types. In CD4^+^ T cell dataset, we applied ISOMAP to detect three hypothesized progression paths spanning across blood and tonsils derived from the naïve T cells (see details in our previously published paper [17]). Diffusion map is a dimensionality reduction algorithm, which captures the non-linear structure of data as a continuum. It demonstrated considerably better performance than the other dimensionality reduction methods PCA or t-SNE for revealing the differentiation structure in single-cell data analysis [21]. In cytofkit, we combined dimensionality reduction methods including ISOMAP and diffusion map with the clustering results to infer inter-subset relatedness, which is expected to help detection of cell differentiation trajectories. Firstly, we down-sampled the number of cells in each cluster to an equal size, thus reducing cell subset density heterogeneities and removing the dominating effect of large populations in the data. Then we ran ISOMAP or diffusion map on the down-sampled dataset and overlaid the clusters onto the transformed dimensions. By checking the median position of clusters in ISOMAP or diffusion map, hypothesized paths of subset progression can be drawn and annotated. The expression profiles of selected markers can be visualized with a Tobit-family generalized linear model (GLM) [30] along the manually defined progression path to either validate the hypothesized path or detect potential progression dynamics.

### Pipeline implementation

**Fig 3:**
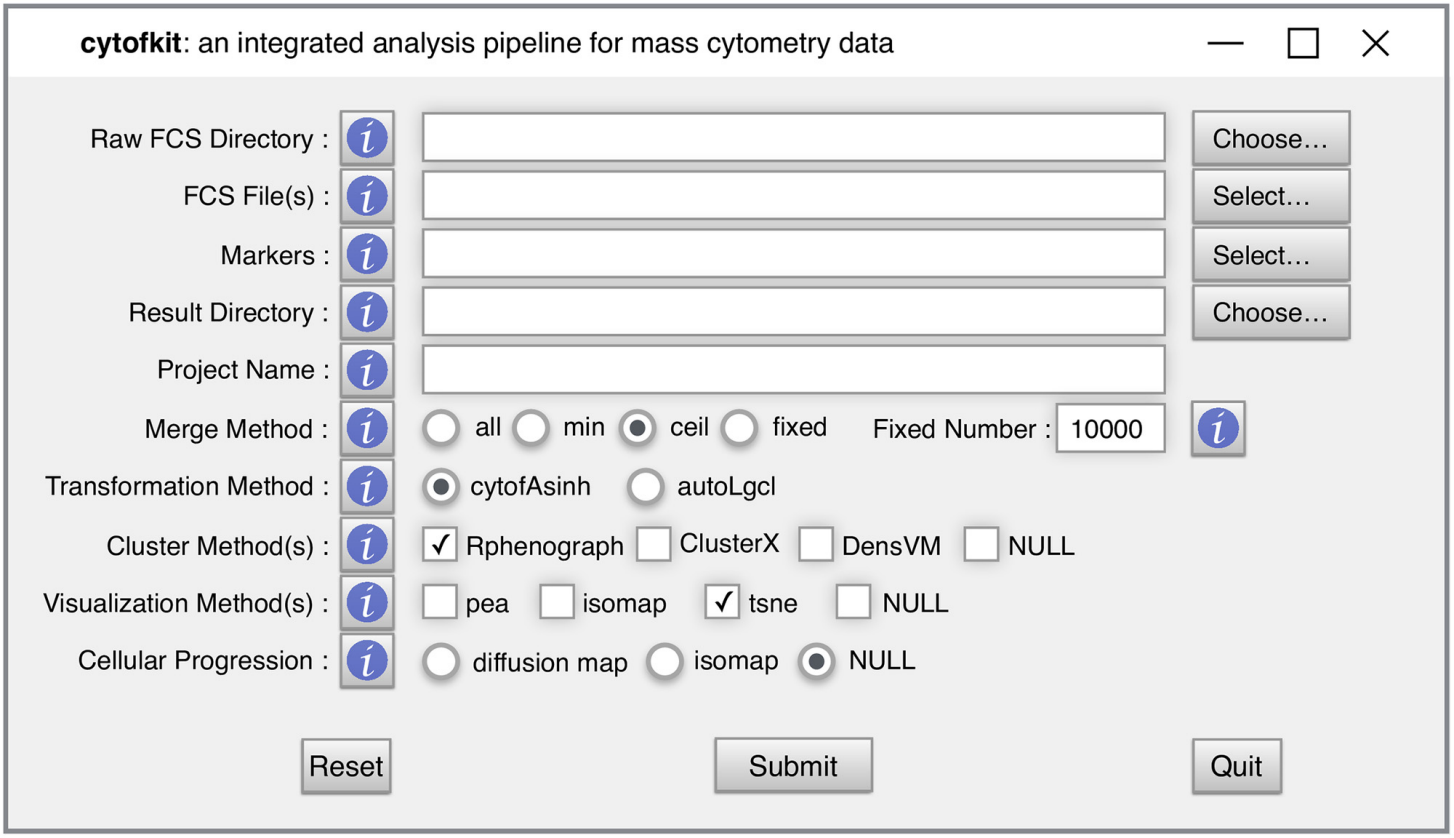
The appearance of the GUI for cytofkit. The GUI provides full options of cytofkit with help buttons explaining the meaning of each parameter.

**Fig 4:**
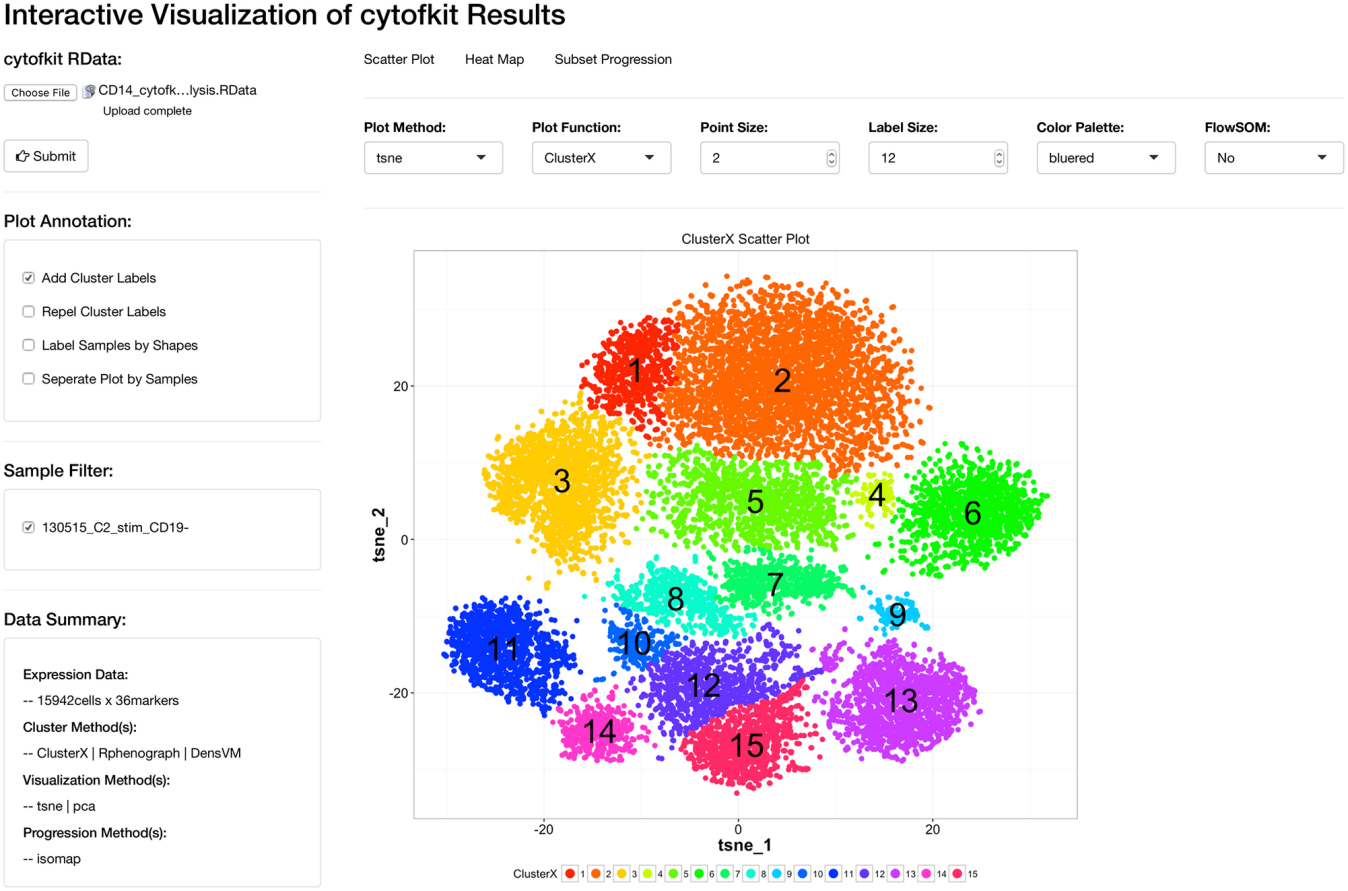
The appearance of the shiny APP for cytofkit. The shiny APP is designed to provide interactively visualization and exploration the cytofkit analysis results. It is integrated into cytofkit package and also a stand-alone online application.

We implemented the cytofkit pipeline in R, and built it as a Bioconductor package (https://bioconductor.org/packages/cytofkit/). ClusterX, as a newly developed clustering algorithm, was implemented as an R package named ClusterX and is available on github (https://github.com/JinmiaoChenLab/ClusterX). PhenoGraph is originally available as python code. We re-implemented the algorithm into an R package named Rphenograph and it is also available on github (https://github.com/JinmiaoChenLab/Rphenograph). ClusterX and Rphenograph are both integrated into the cytofkit package. To facilitate the easy access of cytofkit package, we developed a user-friendly GUI using R tcltk package as shown in Fig 3. To facilitate interactive visualization of the analysis results, the cytofkit package provides a shiny APP which can be deployed locally with function cytofkitShinyAPP(). The analysis results from cytofkit will be saved as an RData object, which can be easily loaded into this shiny APP. This shiny APP provides an interactive interface to visualize and explore the analysis results as shown in Fig 4. In addition, an online version of the shiny APP is also publicly available at https://chenhao.shinyapps.io/cytofkitShinyAPP/. An instruction on usage of the GUI and the package can be found in S2 File as well online in the package vignettes (https://www.bioconductor.org/packages/release/bioc/vignettes/cytofkit/inst/doc/cytofkit_example.html). An instruction on the usage of the shiny APP is included in S3 File as well as online in the package vignette (https://www.bioconductor.org/packages/release/bioc/vignettes/cytofkit/inst/doc/cytofkit_shinyAPP.html). A detailed Rmarkdown file including the analysis procedures and all the data used in the manuscript are available on github (https://github.com/JinmiaoChenLab/cytofkit_analysis_data_code) for reproducing our analysis results. Cytofkit package adds dimensionality reduction and clustering results as additional parameters to the FCS files. Users can open the modified FCS files using other software such as FlowJo to visually verify the clusters with their prior knowledge. They can also overlay manually gated populations onto the t-SNE (ISOMAP, diffusion map) plots; perform manual gating according to the t-SNE plot or clustering results.

## Results and Discussion

We demonstrate the utility of this package using two datasets (included in S1 Dataset). One is a CD14^−^CD19^− ^PBMCs dataset and the other is a CD4^+^ T cell dataset combined from human blood and tonsils. In order to assess the accuracy of cytofkit, we manually gated populations of CD4^+^, CD8^+^, γδT, CD3^+^CD56^+^ NKT and CD3^−^CD56^+^ NK cells from the CD14^−^CD19^−^ PBMCs dataset (gating strategy included in S1 Fig). Populations of naïve (CD45RA^+^CCR7^+^CD45RO^-^), T_H_1 (IFN-γ^+^), T_H_17 (IL-17A^+^) and T_FH_ (CXCR5^hi^PD-1^hi^) cells are manually gated from the CD4^+^ T cell dataset (see in [17]). More information about these two datasets is included in the S1 File data description section.

### Comparison of dimensionality reduction methods for visualization

**Fig 5:**
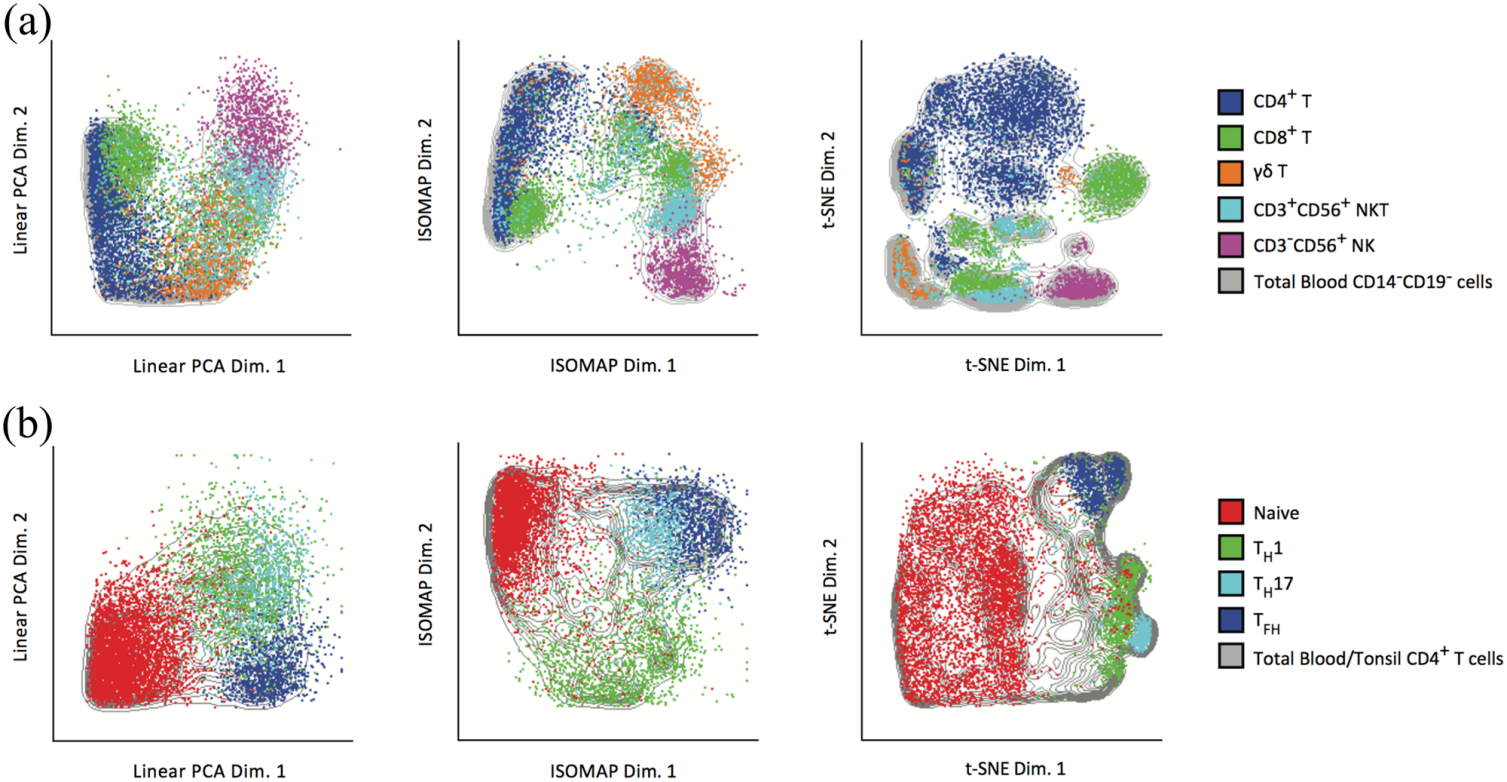
Comparison of dimensionality reduction methods. PCA, ISOMAP and t-SNE are performed on the CD14^−^CD19^−^ PBMCs dataset and the CD4^+^ T cell dataset, respectively. In each panel, Cells are plotted using the first two dimensions of the dimensionality-transformed data and color coded by gated populations. (a) Plot of manually gated CD4^+^, CD8^+^, γδT, CD3^+^CD56^+^ NKT and CD3^−^CD56^+^ NK cell populations from the CD14^−^CD19^−^ PBMCs dataset using PCA, ISOMAP, and t-SNE. (b) Plot of manually gated naïve (CD45RA^+^CCR7^+^CD45RO^-^), T_H_1 (IFN-γ^+^), T_H_17 (IL-17A^+^) and T_FH_ (CXCR5^hi^PD-1^hi^) cell populations from the CD4^+^ T cell dataset using PCA, ISOMAP, and t-SNE.

In order to assess the performance of the three dimensionality reduction methods PCA, ISOMAP and t-SNE, we applied these methods to the above two datasets. For the CD14^−^CD19^−^ PBMCs dataset, we overlaid the gated lymphocyte and NK cell populations onto the plots of the three methods. In Fig 5(a), we observed that PCA displayed a continuous U-shaped pattern of cellular clusters. ISOMAP preserved the U-shaped continuum while showing better resolution of CD4^+^, CD8^+^, γδT, CD3^+^CD56^+^ NKT and CD3^−^CD56^+^ NK cells. The preserved continuum shows the interrelatedness between these subsets. In contrast, t-SNE showed geometrically distinct clusters at much higher resolution and discriminated several populations within the CD4^+^ T cell population. However, we did not observe the continuum as seen with ISOMAP. In the CD4^+^ T cell dataset, after overlaying naïve (CD45RA^+^CCR7^+^CD45RO^−^), T_H_1 (IFN-γ^+^), T_H_17 (IL-17A^+^) and T_FH_ (CXCR5^hi^PD-1^hi^) cells onto the dimensionality-reduced map, we observed that each subset occupied distinct regions in ISOMAP and t-SNE, whereas T_H_1 and T_H_17 cells overlapped in the same region for PCA, as shown in Fig 5(b). Overall, these analyses of two independent datasets highlighted the advantages of non-linear approaches over linear PCA for visualizing and interpreting mass cytometry data.

### Comparison of clustering methods for subset detection

**Fig 6:**
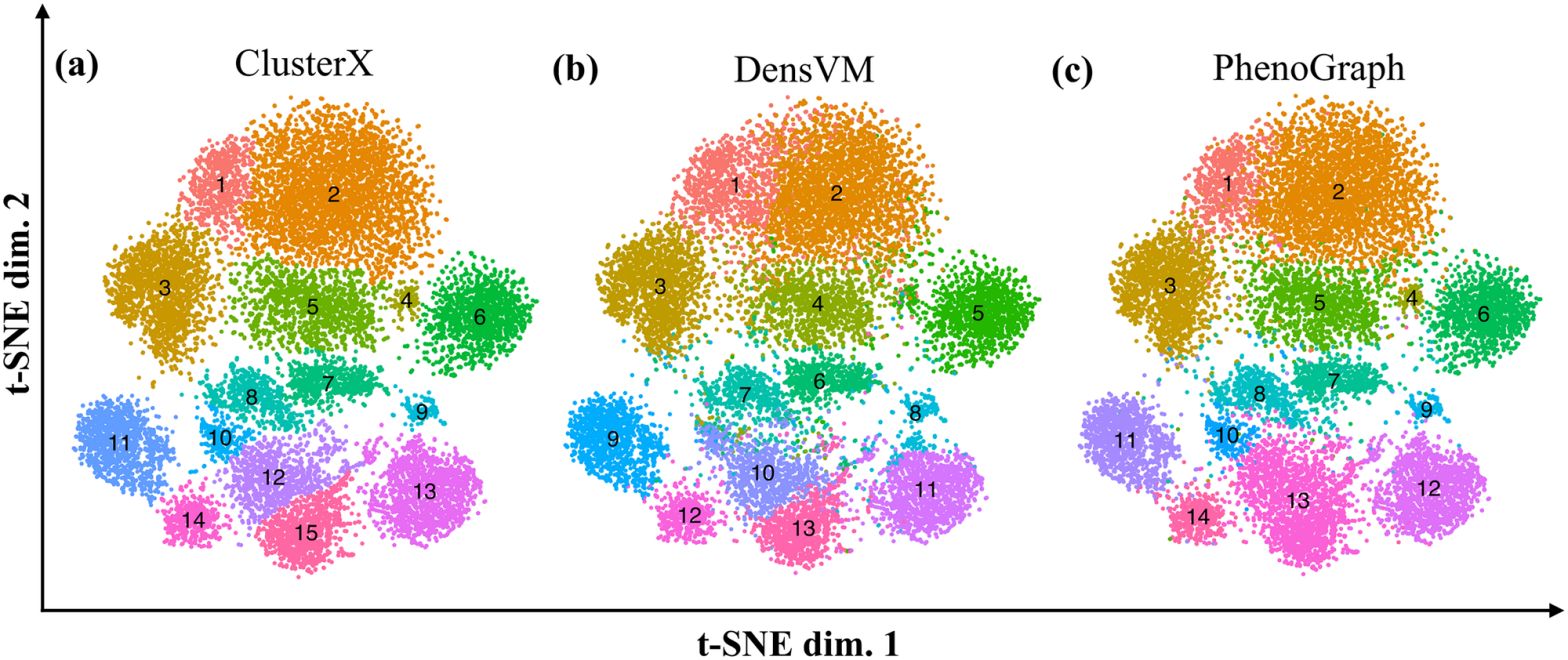
Comparison of clustering methods. Each panel represents one clustering results mapped on the t-SNE plot; from left to right they are (a) ClusterX, (b) DensVM and (c) PhenoGraph. Clusters were annotated by different colors and with cluster ID at the center of the cluster.

Cytofkit contains three clustering methods for automatic subset identification; they are ClusterX, DensVM and PhenoGraph. To assess the performance of these clustering methods, we quantitatively calculated the precision, recall and F-measure of each clustering method, using manually gated populations of CD4^+^, CD8^+^, γδT, NK and NKT cells from the CD14^−^CD19^−^ PBMCs dataset as the gold standard. Fig 6 shows that DensVM detected 13 clusters, PhenoGraph identified 14 clusters and ClusterX 15 clusters. These clusters were mapped to the manually gated populations using FlowJo. As shown in Table 1, ClusterX produced the highest precision in this case; nevertheless, the precision score differences among these three clustering methods are quite small. The F-measures for DensVM, ClusterX and PhenoGraph are 0.886, 0.894 and 0.854 respectively, which shows that all three clustering methods can accurately identify the manually gated cellular populations.

**Table 1:** Precision, recall and F-measure of each clustering method by comparing cluster results to manually gated populations of CD4^+^, CD8^+^, γδT, NK and NKT cells from the CD14^−^CD19^−^ PBMCs dataset.

	Gated Population	Counts	Clusters	Cluster Cell Counts	True Positive	Precesion	Recall	F-measure	Average F-measure
**ClusterX**	CD4	4097	1,2,3,5,10	4265	4029	0.94	0.98	0.96	0.894
	CD8	1897	6,8,12	1881	1702	0.9	0.9	0.9	
	NK	958	9,13	950	934	0.98	0.97	0.97	
	NKT	1302	7,15	1019	840	0.82	0.65	0.73	
	Vd2	1034	4,11,14	1173	1001	0.85	0.97	0.91	
**DensVM**	CD4	4097	1,2,3,4	4108	3898	0.95	0.95	0.95	0.886
	CD8	1897	5,7,10	1992	1710	0.86	0.9	0.88	
	NK	958	8,11	973	947	0.97	0.99	0.98	
	NKT	1302	6,13	1037	863	0.83	0.66	0.74	
	Vd2	1034	9,12	1178	977	0.83	0.94	0.88	
**PhenoGraph**	CD4	4097	1,2,3,5,10	4254	4034	0.95	0.98	0.96	0.854
	CD8	1897	6,8	1375	1269	0.92	0.67	0.78	
	NK	958	9,12	953	936	0.98	0.98	0.98	
	NKT	1302	7,13	1546	917	0.59	0.7	0.64	
	Vd2	1034	4,11,14	1160	998	0.86	0.97	0.91	

**Fig 7:**
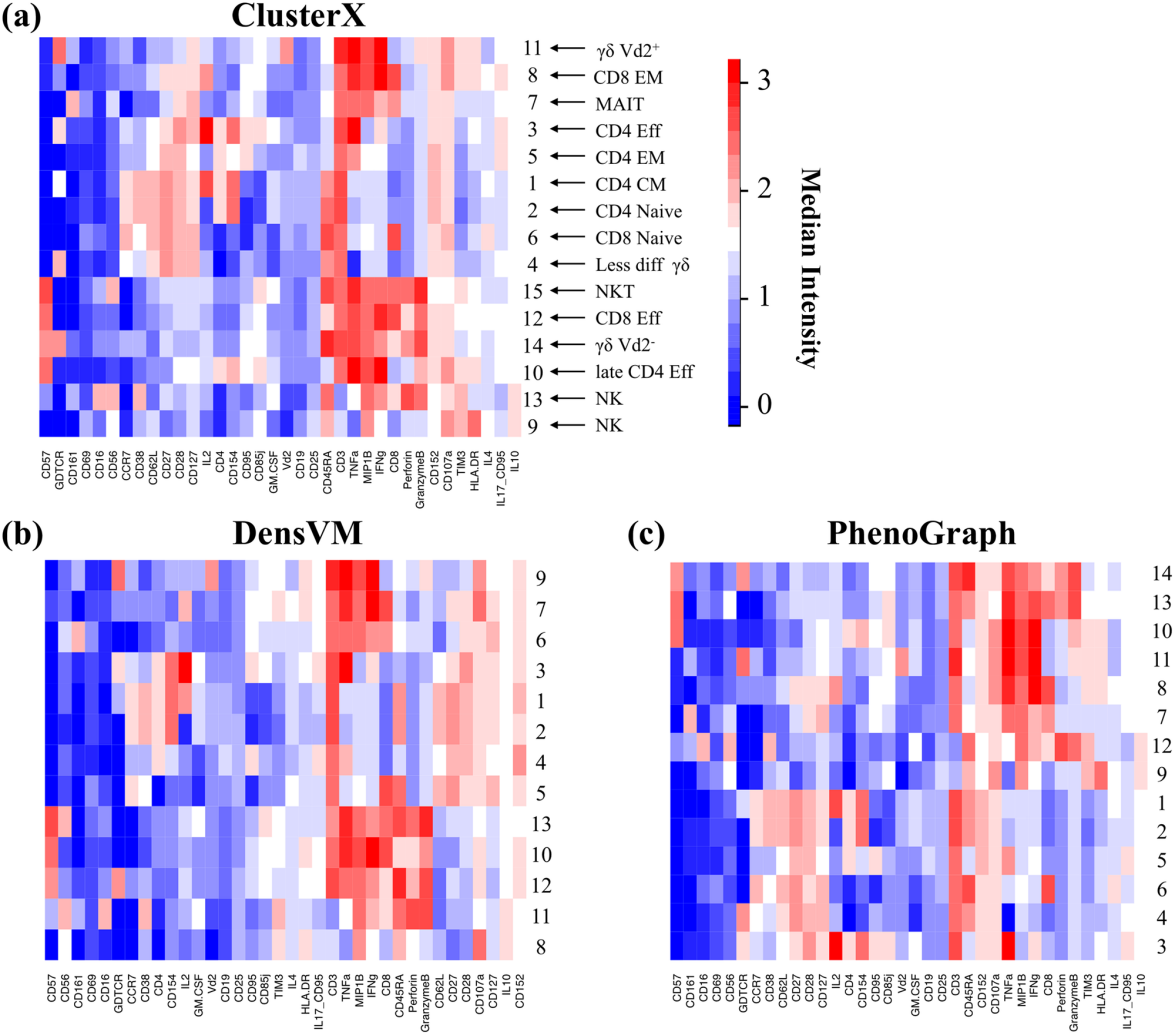
Clusters annotation with heat map. Heat maps show median marker expression of clusters detected by (a) ClusterX, (b) DensVM and (c) PhenoGraph respectively. Heat map row labels represent the cluster IDs and column labels show the marker names. Clusters are annotated by its expression profile in (a).

We annotated the clusters detected by ClusterX based on the median expression of markers, which revealed different stages of CD4^+^ and CD8^+^ T cell differentiation, and three subsets of γδT, NK and NKT cells (Fig 7(a)). Unlike ClusterX, DensVM did not distinguish the CD8 effector population and CD4 late effector population (Fig 7(b)). PhenoGraph detected the ClusterX annotated CD8 effector population and the NKT population as one population (Fig 7(c)). It should be noted that these manually annotated cell populations need to be further validated experimentally. Without experimental validation, we could not determine if clusters 10, 12 and 15 in ClusterX represent truly distinct cell populations or are a result of over-fragmentation. Despite these small differences, all three methods were able to define cellular heterogeneity with a higher efficiency and resolution than manual gating, and we suggest users to try multiple clustering methods for their own data analysis. The clustering results for the CD4^+^ T cell dataset can be seen in S2 Fig.

### Assess ISOMAP, diffusion map and t-SNE for inferring inter-cluster relationship

**Fig 8:**
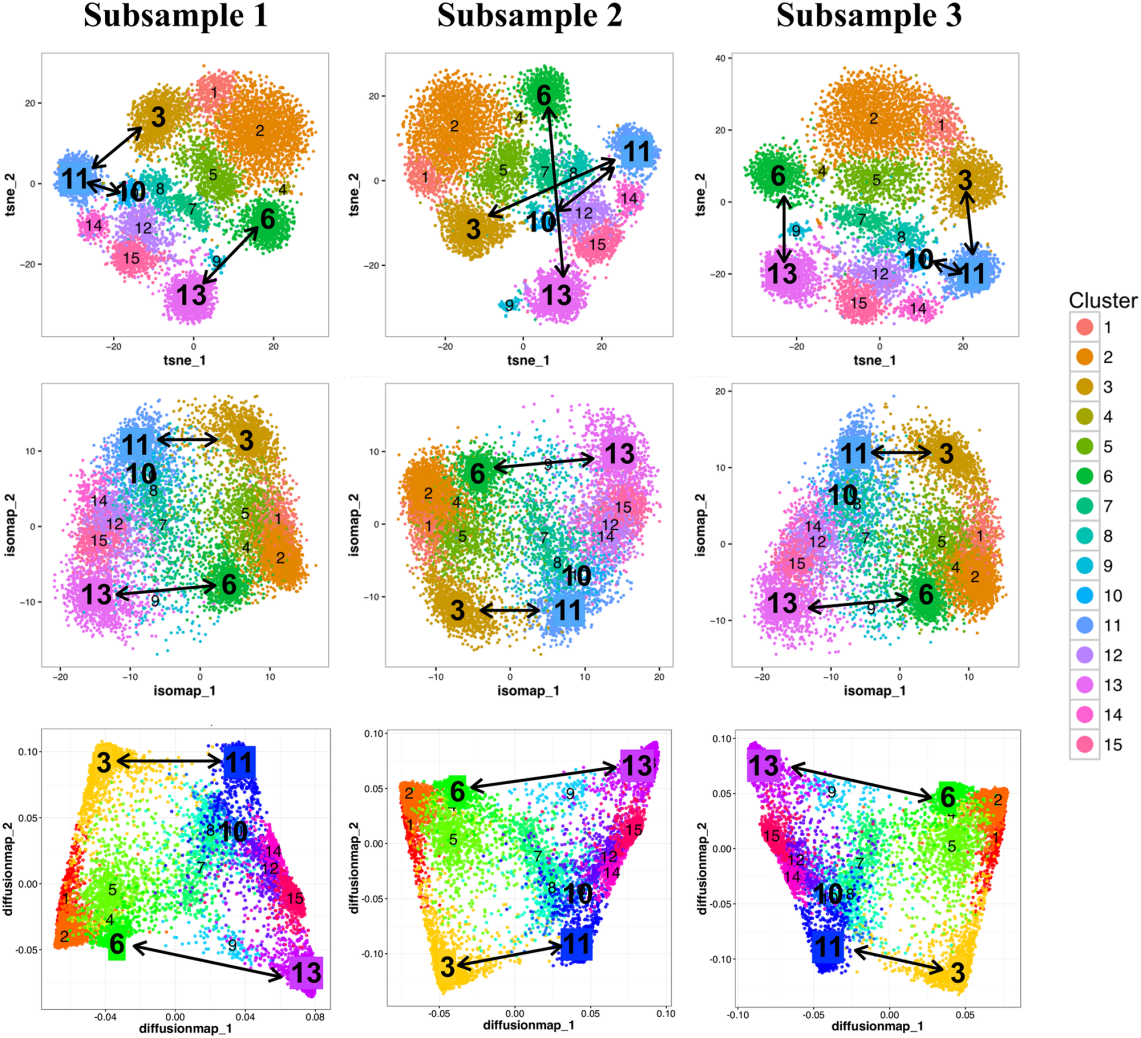
Assessing ISOMAP, diffusion map and t-SNE for inference of subset relationship. Three subsamples are down-sampled from the CD14^−^CD19^−^ PBMCs dataset with equal cell number of 10000. From top to bottom row, the relationship of Cluster X clusters is visualized by t-SNE, ISOMAP and diffusion map on each of the subsample. Cells are color-coded by ClusterX clusters, and cluster IDs are added at the center of each cluster.

To investigate the performance of ISOMAP, diffusion map and t-SNE for mapping potential relationships between cell subsets, we sub-sampled 10000 cells from the CD14^−^CD19^−^ PBMCs dataset and repeated ISOMAP, diffusion map and t-SNE analysis three times. Fig 8 shows that the relative geometric locations of ClusterX clusters on a t-SNE map are a poor measure of between cluster similarities. This is manifested by the evident shift of the relative positions of cell clusters on the t-SNE maps of three subsamples. For example, cluster 11 and cluster 3 were close to each other in subsample 1 and subsample 3 but far apart in subsample 2. Similar changes were also observed on the positional relationships between cluster 11 and 10, or cluster 13 and 6. In contrast, ISOMAP and diffusion map were both able to consistently reproduce the structure of cluster relationship and the relative locations of these clusters remain consistent in all three subsamples.

**Fig 9:**
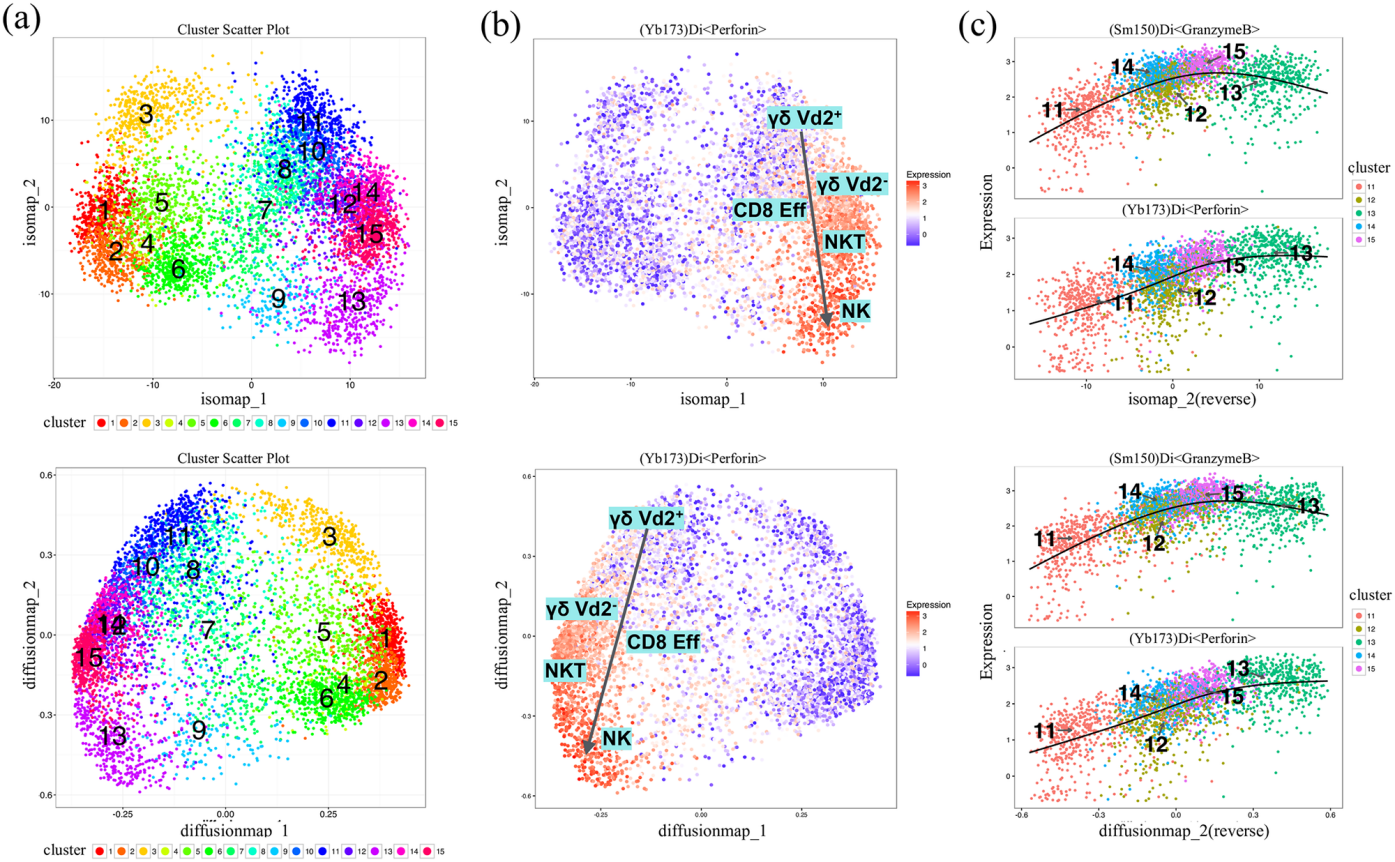
**(a) ISOMAP and diffusion map plots of the down-sampled subsets.** Cells are color-coded by ClusterX clusters. Cluster IDs are labeled at the center of each cluster (b) Plots of the expression level of marker *Perforin* using ISOMAP and diffusion map. Estimated progression among annotated subsets γδ Vd^+^, γδ Vd^−^, CD8 Eff, NKT and NK are added on the plots. (c) The expression profiles of marker *Perforin* and *GranzymeB* for cluster 11, 12, 13, 14 and 15 are visualized on the second component of ISOMAP and diffusion map (reversed order). The regression line estimated using the generalized linear model (GLM) is added for each marker.

To remove the density heterogeneity among cell subsets, we down-sampled 500 cells from each cluster using method *ceil*. Then we plotted the cell subsets using the first two components calculated by ISOMAP and diffusion map (Fig 9). The two methods both give a U-shape like structure of the relationship of cell subsets. On one arm of the U-shape are CD4^+^ and naïve CD8^+^ T cells, which do not exhibit cytotoxic capabilities, as evidenced by the lack of *Perforin* expression (Fig 9(b)). On the opposite arm are γδ Vd^+^, γδ Vd^−^, CD8 Eff, NKT and NK cells, which were located in order along the second component. We found a continuous increase in the expression of *Perforin* and *GranzymeB* along the second component indicating a progression of increased cytotoxic capabilities of these subsets (Fig 9(c)). On another dataset which we previously published, ISOMAP was able to display three hypothesized progression paths of CD4^+^ T cells spanning across blood and tonsils [17]. To summarize, although t-SNE better discriminates cells of distinct phenotypes, we highlight the limitation of t-SNE and suggest using ISOMAP or diffusion map for inferring relatedness between subsets.

## Conclusion

In summary, we developed an integrated analysis pipeline for mass cytometry data, termed cytofkit. Combining state-of-the-art methods and in-house developed algorithms, we aim to provide a one-stop analysis toolkit for mass cytometry data with user-selectable options and customizable framework. Cytofkit can take commands from a user friendly GUI and performs analysis including pre-processing, cell subset detection, plots for visualization and annotation, and inference of the relatedness between cell subsets. In the end, the analysis results can be further explored in an interactive way using the specifically designed shiny APP. Our analytical pipeline provides an automated mass cytometry data analysis toolset, which can be used by bench scientists without any training.

## Availability and Future Directions

Cytofkit was implemented using R and has been published on Bioconductor (https://bioconductor.org/packages/cytofkit/). It is also available on github (https://github.com/JinmiaoChenLab/cytofkit). Detailed documentations and demos can be found in the vignettes of the package, including cytofkit quick start (https://www.bioconductor.org/packages/release/bioc/vignettes/cytofkit/inst/doc/cytofkit_example.html), cytofkit workflow (https://www.bioconductor.org/packages/release/bioc/vignettes/cytofkit/inst/doc/cytofkit_workflow.html) and cytofkit shinyAPP (https://www.bioconductor.org/packages/release/bioc/vignettes/cytofkit/inst/doc/cytofkit_shinyAPP.html). Cytofkit is developed with a general framework, which makes it easily extensible to add in new methods and also applicable to other multi-parameter data types. We are continually working on new algorithms for inferring cellular progression as well as meta-clustering methods for comparative analysis between multiple batches of data. New methods will be added into cytofkit to make it more useful for automatic mass cytometry data analysis.

## Supporting Information

S1 Dataset Zip file containing cytofkit package source code, the CD14^−^CD19^−^ PBMCs dataset and the CD4^+^ T cell dataset. (ZIP)

S1 File Data description, data transformation methods, detailed description of ClusterX and analysis procedure and codes. (DOCX)

S2 File Instruction on the usage of the cytofkit GUI and package. (DOCX)

S3 File Instruction on the usage of cytofkit shiny APP. (DOCX)

S1 Fig Gating strategy for CD14^−^CD19^−^ PBMCs dataset. Five populations including CD4^+^, CD8^+^, γδT, CD3^+^CD56^+^ NKT and CD3^−^CD56^+^ NK cells are manually gated from the CD14^−^CD19^−^ PBMCs dataset using FlowJo software. (TIF)

S2 Fig The comparison of clustering results for CD4^+^ T cell dataset. Each panel represents one clustering results mapped on the t-SNE plot; from left to right they are (a) clustering results of ClusterX, (b) clustering results of DensVM and (c) clustering results of PhenoGraph. Clusters were annotated by different colors and with cluster ID at the center of the cluster.
(TIF)

S3 Fig Illustration of density peak detection in ClusterX using R15 dataset. (a) Scatter plot of the D15 dataset with 15 clusters, clusters are color labeled and cluster centers are labeled by circles with crosses. (b) CFSFDP’s density peak detection method in which plots of delta against rho are generated, and users manually set a threshold point to determine the density peaks (c) ClusterX’s density peak detection method in which plots of sigma against the rank of rho are generated, and true peak points have significantly higher values of sigma. (d) ClusterX uses the generalized ESD to detect the density peaks automatically, wherein sigma is assumed to have normal distribution and peaks are regarded as anomalies that have significantly higher sigma values.
(TIF)

S4 Fig Robustness of peak number with *p*-value selection in ClusterX. The number of density peaks is plotted over different α values within the range from 0.001 to 0.05 on the R15 datasets.
(TIF)

S5 Fig Split-apply-combine implementation in ClusterX. In ClusterX, data are first split row-wisely into chunks, the distance matrix is calculated in each chunk to be restricted in a limited size; then apply the calculation function for each parameters in each chunk; Finally the parameters are combined from all chunks for post processing.
(TIF)
