# Supplementary material for: Cytofkit: A Bioconductor Package for an Integrated Mass Cytometry Data Analysis Pipeline
Source: PLoS Comput Biol. 2016 Sep 23;12(9):e1005112. doi: 10.1371/journal.pcbi.1005112 (PMC5035035; doi:10.1371/journal.pcbi.1005112)
Supplement: S1 File — (DOCX) [file pcbi.1005112.s002.docx]

# Supporting Information for “Cytofkit: A Bioconductor Package for an Integrated Mass Cytometry Data Analysis Pipeline”

**Authors**

Hao Chen, Mai Chan Lau, Michael Wong, Evan Newell, Michael Poidinger, Jinmiao Chen

**Affiliations**

Agency for Science, Technology and Research (A*STAR), Singapore Immunology Network (SIgN), Singapore 138648

**Supporting file S1**

**Data Description**

CD14^−^CD19^−^ PBMCs dataset was derived from a Hepatitis B seropositive donor. Cells were stimulated with PMA and ionomycin in the presence of Brefeldin A (BFA) and monensin for 4 hours and then stained with a panel of 40 markers, including antibodies that measured different lineage populations, differentiation/activation status and function. CD14^+^ monocytes and CD19^+^ B cells were gated out. CD4^+^ T cell dataset contains cells from 4 PBMC and 3 tonsil donors. Cells were stained with a unique panel of 40 markers primarily found on CD4^+^ T cells, including antibodies that measured differentiation/activation status, trafficking receptors and function.

The staining panels for these two mass cytometry datasets are shown in **Table A** below:

| **Table A: Staining panels for mass cytometry datasets** | | | | | | | | |
| --- | --- | --- | --- | --- | --- | --- | --- | --- |
|  |  | **Panel 1: CD14^−^CD19^−^ from PBMCs** | | |  | **Panel 2: CD4^+^ from PBMCs and Tonsils** | | |
| **Metal** |  | **Antibody** | **Clone** | **Company** |  | **Antibody** | **Clone** | **Company** |
| 112/114 |  | **Qdot800-CD14** | TuK4 | Molecular Probes |  | **Qdot800-CD14** | TuK4 | Molecular Probes |
| 115 |  | **CD57** | HCD57 | Biolegend |  | **CD57** | HCD57 | Biolegend |
| 139 |  | **CD45** | HI30 | Biolegend |  | **CD45** | HI30 | Biolegend |
| 140 |  | **CD5** | UCHT2 | Biolegend |  | **CD5** | UCHT2 | Biolegend |
| 141 |  | **IFN-γ** | 4S.B3 | eBioscience |  | **FITC-CLA (primary) anti-FITC (secondary)** | HECA-452 FIT-22 | Biolegend |
| 142 |  | **HLA-DR** | L243 | Biolegend |  | **HLA-DR** | L243 | Biolegend |
| 143 |  | **CD62L** | DREG56 | BD Bioscience |  | **IFN-γ** | 4S.B3 | eBioscience |
| 144 |  | **CD69** | FN50 | Biolegend |  | **CD69** | FN50 | Biolegend |
| 145 |  | **CD16** | 3G8 | Biolegend |  | **CD27** | LG.7F9 | eBioscience |
| 146 |  | **CD8α** | SK1 | Biolegend |  | **CD8α** | SK1 | Biolegend |
| 147 |  | **TNF-α** | Mab11 | eBioscience |  | **TNF-α** | Mab11 | eBioscience |
| 148 |  | **CD85j** | 292319 | R&D Systems |  | **CCR4** | 205410 | R&D Systems |
| 149 |  | **CD4** | SK3 | Biolegend |  | **CD4** | SK3 | Biolegend |
| 150 |  | **Granzyme B** | CLB-GB11 | Abcam |  | **Granzyme B** | CLB-GB11 | Abcam |
| 151 |  | **CD27** | LG.7F9 | eBioscience |  | **IL-2** | MQ1-17H12 | eBioscience |
| 152 |  | **Vδ2** | B6 | Biolegend |  | **CD45RO** | UCHL1 | Biolegend |
| 153 |  | **CD107a** | H4A3 | BD Bioscience |  | **CD107a** | H4A3 | BD Bioscience |
| 154 |  | **CD3** | UCHL1 | Biolegend |  | **CD3** | UCHL1 | Biolegend |
| 155 |  | **CTLA-4** | BNI3 | BD Bioscience |  | **CTLA-4** | BNI3 | BD Bioscience |
| 156 |  | **CD19** | HIB19 | Biolegend |  | **CD19** | HIB19 | Biolegend |
| 157 |  | **TIM-3** | A11 | In house (SIgN) |  | **Biotin-Foxp3(primary)** | PCH101 | eBioscience |
| 158 |  | **CD56** | NCAM16.2 | BD Bioscience |  | **CD56** | NCAM16.2 | BD Bioscience |
| 159 |  | **Biotin-IL-10 (primary)** | JES3-12G8 | Biolegend |  | **CD161** | HP-3G10 | Biolegend |
| 160 |  | **CD28** | CD28.2 | Biolegend |  | **IL-17F** | SHLR17 | eBioscience |
| 161 |  | **CD38** | HIT2 | Biolegend |  | **CD38** | HIT2 | Biolegend |
| 162 |  | **IL-4** | MP4-25D2 | Biolegend |  | **IL-4** | MP4-25D2 | Biolegend |
| 163 |  | **CD127** | A019D5 | Biolegend |  | **CD45RA** | HI100 | BD Bioscience |
| 164 |  | **IL-17A** | BL168 | Biolegend |  | **CD25** | M-A251 | BD Bioscience |
| 165 |  | **CD95** | DX2 | Biolegend |  | **CD40L** | 24-31 | eBioscience |
| 166 |  | **IL-2** | MQ1-17H12 | eBioscience |  | **CXCR5** | RF8B2 | BD Bioscience |
| 167 |  | **PE-γδTCR (primary) anti-PE (secondary)** | SA6.E9 PE001 | Invitrogen Biolegend |  | **PD-1** | eBioJ10s | eBioscience |
| 168 |  | **CCR7** | 150503 | R&D Systems |  | **IL-22** | Poly5161 | Biolegend |
| 169 |  | **CD25** | M-A251 | BD Bioscience |  | **ICOS** | C398.4A | Biolegend |
| 170 |  | **APC-CD161 (primary) anti-APC (secondary)** | 191B8 APC003 | Miltenyi Biotec Biolegend |  | **IL-10** | 3BB2 | Abnova |
| 171 |  | **CD40L** | 24-31 | eBioscience |  | **CCR6** | G034E3 | Biolegend |
| 172 |  | **GM-CSF** | BVD2-21C11 | Biolegend |  | **GM-CSF** | BVD2-21C11 | Biolegend |
| 173 |  | **Perforin** | B-D48 | Abcam |  | **CCR7** | 150503 | R&D Systems |
| 174 |  | **CD45RA** | HI100 | BD Bioscience |  | **IL-13** | 5G5 | Abnova |
| 175 |  | **Mip-1β** | D21-1351 | BD Bioscience |  | **IL-9** | MH9A4 | Biolegend |
| 176 |  | **Vα24Jα18** | 6B11 | Biolegend |  | **IL-17A** | BL168 | Biolegend |
|  |  |  |  |  |  |  |  |  |
|  |  | \| 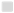 Intracellular antibodies \| \| --- \| | |  |  |  |  |  |

Markers used in CD14^−^CD19^−^ PBMCs dataset for analysis:

CD57, IFNg, HLA-DR, CD62L, CD69, CD16, CD8, TNFa, CD85j, CD4, GranzymeB, CD27, Vd2, CD107a, CD3, CD152, CD19, TIM3, CD56, IL10, CD28, CD38, IL4, CD127, IL17_CD95, CD95, IL2, GDTCR, CCR7, CD25, CD161, CD154, GM-CSF, Perforin, CD45RA, MIP1B

Markers used in CD4^+^ T cell dataset for analysis:

CCR4, CCR6, CCR7, CD107a, CD161, CD25, CD27, CD38, CD40L, CD45RA, CD45RO, CD57, CD69, CLA, CTLA-4, CXCR5, Foxp3, GM-CSF, GranzymeB, HLA-DR, ICOS, IFN-g, IL-10, IL-13, IL-17A, IL-17F, IL-2, IL-22, IL-4, IL-9, PD-1, TNF-a

**Data transformation methods**

Cytofkit provides four transformation methods, including arcsinh, cytofAsinh, logicle and autoLgcl. arcsinh is the inverse hyperbolic sine transformation (arcsinh) with a cofactor of 5, which is commonly used for mass cytometry data transformation. However, mass cytometry data usually contains negative values introduced artificially by the CyTOF acquisition software, which doesn’t carry biological information. So we suggest a modified arcsinh transformation termed cytofAsinh for mass cytometry data analysis. Using cytofAsinh, negative values will be replaced with random values sampled from a normal distribution with mean of 0 and standard derivation of 0.01. R codes of cytofAshinh used in cytofkit is shown below:

cytofAsinh <- function(value, cofactor = 5) {

value <- value-1

loID <- which(value < 0)

if(length(loID) > 0)

value[loID] <- rnorm(length(loID), mean = 0, sd = 0.01)

value <- value / cofactor

value <- asinh(value)

return(value)

}

Logicle transformation is widely applied for flow cytometry data transformation. With logicle option, values around zero are scaled linearly, while higher intensity values are scaled in a logarithmic fashion. The transition from linear to logarithmic scaling in logicle transformation is determined by the parameter ω of the formula. As suggested in paper [1], the fifth percentile of the negative values is set as the reference value *r* to estimate the ω. However this estimation fails when there exist many extreme negative values in the data. We addressed this limitation by performing a negative outliers filtering before calculating the *r* using the fifth percentile of the negative values, which is introduced in [2], called autoLgcl. R codes for autoLgcl is included in cytofkit package, which can be checked with command cytofkit:::autoLgcl.

We provide the users with multiple options for data transformation. We would like to point out that the optimal transformation method is data-dependent [3], and there is no best transformation for all cases.

**Detailed description of ClusterX**

Here we describe our ClusterX workflow for mass cytometry data clustering in four steps as illustrated in **Fig 2(a)**. (i) The first step is applying t-SNE to embed the cells into two-dimensional space [4]. (ii) The second step is to estimate local density on the t-SNE map. We applied an exponential kernel to estimate the local density of each data point as illustrated in **Fig 2(b)**. For data point i, the local density is defined by

$$\rho_{i}=\sum_{j:j\neq i} e^{-{(\frac{d_{ij}}{d_{c}})}^{2}}$$

Where d_ij_ is the Euclidean distance between data point i and j, and d_c_ is the kernel bandwidth. Points closer to point i contribute more to the local density of point i, while points with higher distance contribute less. We adopted the strategy for d_c_ determination as in Rodriguez et al. [5] to select the d_c_ that can make the average local density of all points fall within the 1-2 percentile range of the total number of points. (iii) The third step is to automatically detect the density peaks which represent the cluster centers and this is illustrated using the R15 dataset [6] in **S3 Fig**. Here we define another parameter of point i called δ_i_, which is its minimum distance to any other points with higher density as below

$$\delta_{i}=\left\{ \begin{aligned} \min_{j:j\neq i, \rho_{j}>\rho_{i}} \left( d_{ij} \right) , if exists\rho_{j}>\rho_{i} \\ \max_{j:j\neq i} \left( d_{ij} \right), if not exists \rho_{j}>\rho_{i} \end{aligned} \right.$$

The assumption for the density peaks in the CFSFDP algorithm is that a density peak should have high local density ρ_i_, and relatively large distance to the nearest point with a higher local density, which is defined by δ_i_. This is implemented in CFSFDP by plotting all δ_i_ against ρ_i_, and then requiring a manual selection of thresholds to determine the peak points as in **S3 Fig (b)**. In ClusterX, we automate this step of detecting those density peaks with high ρ_i_ and anomalously large δ_i_. First we combine the local density ρ_i_ and distance δ_i_ into one parameter θ_i_ as

$$\theta_{i}=\rho_{i}\times\delta_{i}$$

With this transformation, the points with relatively high δ_i_ but low ρ_i_ will be assigned a low value of θ_i_, while those points with both high δ_i_ and high ρ_i_ will be assigned a large value of θ_i_. Thus peak detection can be performed by looking for anomalously high values of θ_i_. Here we use the generalized ESD method, which tests for outliers or anomalies to automatically detect such points with anomalously large θ_i_ as pseudo codes below

$$\begin{matrix} peakID=() \\ j=0 \\ while\left( peakExist=TRUE \right) \\ R= \frac{max(\theta_{i})-\bar{\theta}}{s}; \\ Rid=\left( which (\theta_{i}=\max\left( \theta_{i} \right) \right); \\ p=1-\frac{\alpha}{2\left( n-j-1 \right)}; \\ \lambda=\frac{(n-j)t_{p,n-j-1}}{(n-j-1+t_{p,n-j-1}^{2})(n-j+1)}; \\ if (R>\lambda) \\ add Rid to peakID; \\ remove \theta_{Rid} from \theta; \\ j=j+1; \\ else \\ peakExist=FALSE; \end{matrix}$$

Where $\bar{\theta}$ and s denote the mean and standard deviation of θ_i_ respectively; t_p,v_ is the 100p percentage point from the t-distribution with v degrees of freedom; and α represents the significant level of the data point being identified as outliers. In regions where ρ_i_ is high, cells with low δ_i_ will be assigned with high θ_i_. Therefore we might detect false cluster centers by just checking θ_i_. Thus we apply the same generalized ESD method to check for anomalous values of δ_i_ as well. Then, the cluster centers are determined by the intersection of the two sets of anomalies determined from both θ_i_ and δ_i_. In our method, the number of density peaks may depend on the pre-selected significance level α. However, when tested on the R15 datasets, the number of density peaks is robust over different α values within the range from 0.001 to 0.05 (**S4 Fig**). (iv) The last step is the cluster assignment as shown in **Fig 2(c)**. We followed the strategy used in CFSFDP, which assigns the clusters by taking both the distance and density of the nearest neighbor into consideration. Firstly, density peaks detected from step (iii) are identified as cluster centers and assigned a unique cluster ID. Then each remaining point is assigned to its nearest density peak as follow

$$C_{i}=\left\{ \begin{aligned} rank of i in peakID; if &i\in peakID \\ C_{j}, j:\rho_{j}>\rho_{i}& \wedge d_{ij}=\min_{k:k\neq i} d_{ik}; if &i\notin peakID \end{aligned} \right.$$

ClusterX is also designed to handle large datasets. One basic step of ClusterX is the calculation of cell-cell distance matrix d_ij_. Calculating the entire dissimilarity matrix d_ij_ for large number of cells is memory intensive. For millions of cells, the size of dissimilarity matrix goes up to 10 and more gigabits, which is beyond the capacity of current PCs. To make ClusterX method applicable on big datasets, we apply a split-apply-combine strategy [7]. As illustrated in **S5 Fig**, we first partition the data into chunks and then calculate the dissimilarity matrix for each chunk. In addition to the two key parameters ρ_i_ and δ_i_, a new parameter called link-cell ID is calculated for each chunk. The link-cell ID for one cell is the ID of its nearest cell that has higher density, which avoids duplicate calculations in the clustering assigning step. Then the parameters including ρ_i_, δ_i_ and link-cell ID of each cell calculated for each chunk is combined for density peak detection and cluster assigning. This split-apply-combine strategy implemented in ClusterX addresses the big-memory issue and also makes the algorithm available for parallel computing for further performance enhancement.

We further validated ClusterX on two mass cytometry benchmark datasets (Levine_2015_marrow_32 and Levine_2015_marrow_13, available from FlowRepository (FR-FCM-ZZPH)) used in paper [8]. We run ClusterX on these two datasets using all the markers, and tested the performance using evaluation scripts provided on github (<https://github.com/lmweber/cytometry-clustering-comparison>). For Levine_2015_marrow_32 dataset, ClusterX results have mean F1 score of 0.674, which was ranked between PhenoGraph(0.689) and ClusterX(0.599). And for Levine_2015_marrow_13 dataset, ClusterX ranked second with mean F1 score of 0.569, slightly behind FlowSOM_meta(0.593). Analysis codes are provided on github (https://github.com/JinmiaoChenLab/cytofkit_analysis_data_code)

**Analysis procedure and codes**

The manual gating is performed using FlowJo V10.0.8. Most of the analysis performed in the manuscript is done using cytofkit version 1.2.1, including data transformation, dimensionality reduction and clustering analysis. The inter-subset relatedness analysis is performed using cytofkit version 1.4.8.

The analysis codes and data are provided in github for reproducible research (<https://github.com/JinmiaoChenLab/cytofkit_analysis_data_code>). Briefly, for the CD14^−^CD19^−^ PBMCs dataset (130515_C2_stim_CD19-.fcs), we firstly extracted the expression value of all cells for selected markers listed in the above **Data Description** section; data were transformed using the autoLgcl method. Then dimensionality reduction was performed using method PCA, ISOMAP and t-SNE. Clustering analysis was conducted with method ClusterX, PhenoGraph and DensVM. The cluster results from ClusterX are manually annotated according to the median expression level of markers in each cluster, which is represented on the heat map of median expression values. Cluster results were quantitatively evaluated by F1 measure using the manual gating as benchmark. Then three subsamples were randomly drawn from the expression matrix with an equal size of 10000 cells to assess the performance of ISOMAP, diffusion map and t-SNE for inferring inter-cluster relationship. After that, a cluster-based down sampling was adopted to remove the density heterogeneity among clusters. Specifically, 500 cells were sampled from each cluster using *ceil* method and then combined for estimating subset relationship using ISOMAP and diffusion map. A hypothesized progression path was drawn from both the ISOMAP and diffusion map plot. And the expression of marker *Perforin* and *GranzymeB* along the hypothesized path were explored using a Tobit-family generalized linear model. For the CD4^+^ T cell dataset (S1 PBMC.fcs, S2 PBMC.fcs, S3 PBMC.fcs, S4 PBMC.fcs, S5 Tonsil.fcs, S6 Tonsil.fcs, S7 Tonsil.fcs), 10000 cells were sampled from each FCS file using the *ceil* method with autoLgcl transformation and then combined into one expression matrix. PCA, ISOMAP and t-SNE were tested on this matrix. Clustering analysis was performed using ClusterX, DensVM and PhenoGraph respectively. Further analysis procedures on this dataset was included in previous publication [9].

**Reference**

1. Parks DR, Roederer M, Moore WA. A new “logicle” display method avoids deceptive effects of logarithmic scaling for low signals and compensated data. Cytom Part A. 2006;69: 541–551.

2. Monaco G, Chen H, Poidinger M, Chen J, de Magalhaes JP, Larbi A. flowAI: automatic and interactive anomaly discerning tools for flow cytometry data. Bioinformatics. 2016; btw191–. doi:10.1093/bioinformatics/btw191

3. Finak G, Perez J, Weng A, Gottardo R. Optimizing transformations for automated, high throughput analysis of flow cytometry data. BMC Bioinformatics. 2010;11: 546. doi:10.1186/1471-2105-11-546

4. Maaten L Van Der, Hinton G. Visualizing Data using t-SNE. J Mach Learn Res. 2008;9: 2579–2605.

5. Rodriguez A, Laio A. Clustering by fast search and find of density peaks. Science. 2014;344: 1492–6. doi:10.1126/science.1242072

6. Veenman CJ, Reinders MJT, Backer E. A maximum variance cluster algorithm. IEEE Trans Pattern Anal Mach Intell. IEEE; 2002;24: 1273–1280. doi:10.1109/TPAMI.2002.1033218

7. Wickham H. The Split-Apply-Combine Strategy for Data. J Stat Softw. 2011;40: 1–29. doi:10.1.1.182.5667

8. Weber LM, Robinson MD. Comparison of Clustering Methods for High-Dimensional Single-Cell Flow and Mass Cytometry Data [Internet]. bioRxiv. 2016. doi:10.1101/047613

9. Wong MT, Chen J, Narayanan S, Lin W, Anicete R, Kiaang HTK, et al. Mapping the Diversity of Follicular Helper T Cells in Human Blood and Tonsils Using High-Dimensional Mass Cytometry Analysis. Cell Rep. Elsevier; 2016;11: 1822–1833. doi:10.1016/j.celrep.2015.05.022
