# Supplementary material for: Cytofkit: A Bioconductor Package for an Integrated Mass Cytometry Data Analysis Pipeline
Source: PLoS Comput Biol. 2016 Sep 23;12(9):e1005112. doi: 10.1371/journal.pcbi.1005112 (PMC5035035; doi:10.1371/journal.pcbi.1005112)
Supplement: S2 File — (DOCX) [file pcbi.1005112.s003.docx]

cytofkit: Quick Start

Table of Contents

Install and Load the Package 1

Options for Using cytofkit Package 2

Run with GUI 2

Run with the Core Function 3

Run with Commands (Step-by-Step) 4

Pre-processing 4

Cell Subset Detection 5

Cell Subset Visualization and Interpretation 6

Inference of Subset Progression 12

Post-processing 20

Session Information 20

# Install and Load the Package

To install **cytofkit** package, start R and run the following codes on R console:

source("https://bioconductor.org/biocLite.R")
biocLite("cytofkit")

Notes: **cytofkit** GUI is dependent on XQuartz windowing system (X Windows) on Mac (OS X > 10.7). Install XQuartz from <http://xquartz.macosforge.org>.

- Download the disk image (dmg) file for [XQuartz](https://dl.bintray.com/xquartz/downloads/XQuartz-2.7.9.dmg).
- Double-clicking on the file to start the installation, you can do it safely by clicking through all the defaults.
- After the installer runs, you'll have to log out and back on to your Mac OS X account.

Load the Package:

library("cytofkit")

Read the package description:

?cytofkit-package

# Options for Using cytofkit Package

**cytofkit** provides three ways to employ the workforce of this package:

## Run with GUI

The easiest way to use **cytofkit** package is through the GUI. The GUI provides all main options of **cytofkit** on a visual interface. To launch the GUI, load the package and type the following command:

cytofkit_GUI()

The interface will appear like below, you can click the information button **!** to check the explanation for each entry and customize your own analysis.

On Windows:


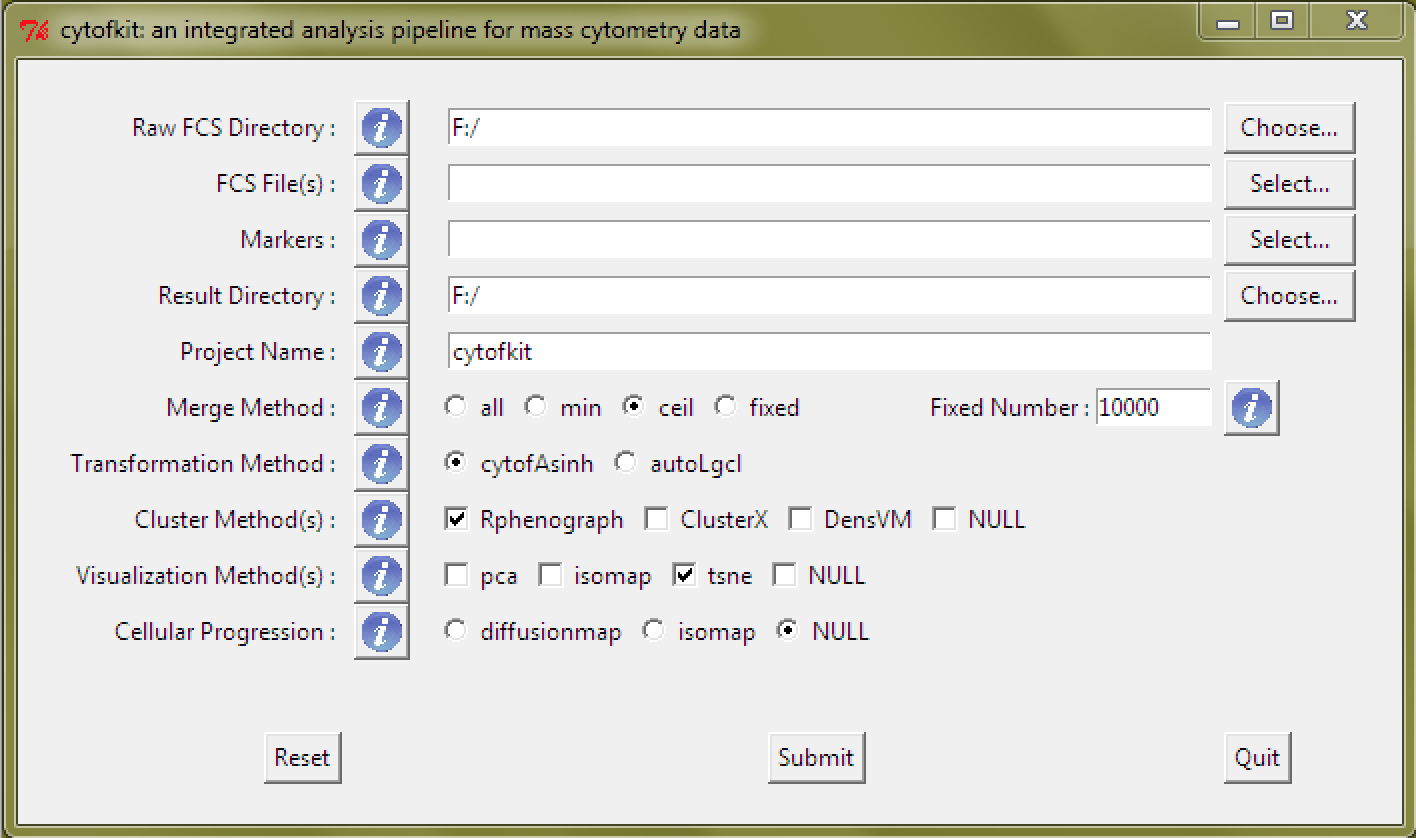


cytofkit GUI

On Mac:


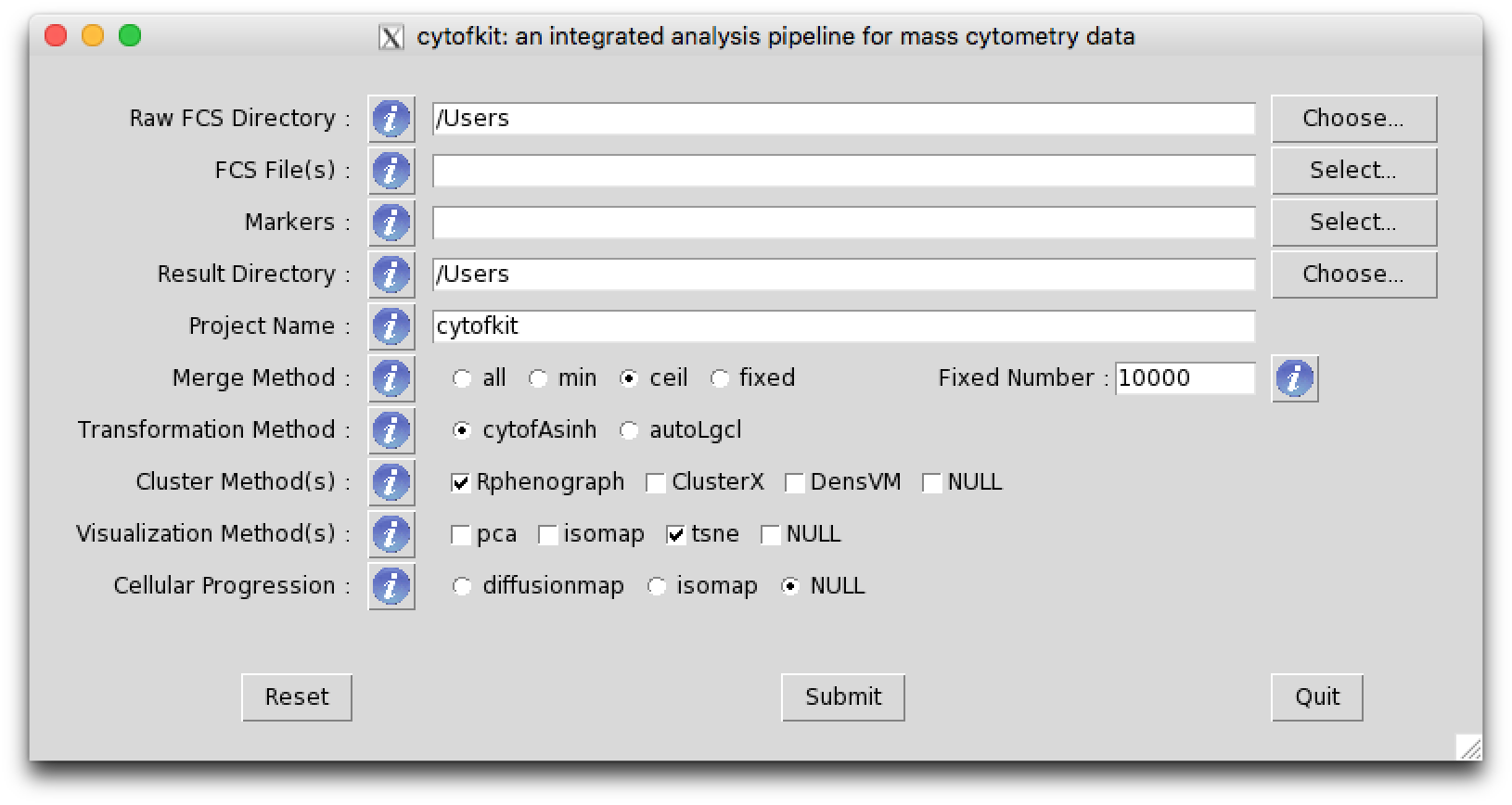


cytofkit GUI

Start your analysis as simply as following:

- Choose the input fcs files from the directory where you store FCS data;
- Select the markers from the auto-generated marker list;
- Choose the directory where to save your output;
- Give a project name as a prefix for the names of result files;
- Select a data merging method if you have multiple FCS files;
- Select your clustering method(s), visualization method(s), progression estimation method(s)

Then submit it, that's all.

Depends on the size of your data, it will take some time to run the analysis. Once done, a window will pop up, showing you the path where the results have been stored, and asking you if open the shiny web APP. If YES, the shiny APP will be deployed locally and opened in your default web browser. Among the saved results, a special R data object with suffix of ***.RData*** is for loading the results into the shiny APP. Choose the ***.RData*** file on the shiny APP then submit it, your journey of exploring the reulsts starts.

## Run with the Core Function

**Cytofkit** provides a core function cytofkit() to drive the analysis pipeline of mass cytometry data. Users only need to define several key parameters to make their analysis automatically. One simple example of running cytofkit using the core function is like this:

set.seed(100)
dir <- system.file('extdata',package='cytofkit')
file <- list.files(dir ,pattern='.fcs$', full=TRUE)
parameters <- list.files(dir, pattern='.txt$', full=TRUE)
res <- cytofkit(fcsFiles = file,
 markers = parameters,
 projectName = 'cytofkit_test',
 transformMethod = "cytofAsinh",
 mergeMethod = "all",
 dimReductionMethod = "tsne",
 clusterMethods = c("Rphenograph", "ClusterX"), ## accept multiple methods
 visualizationMethods = c("tsne", "pca"), ## accept multiple methods
 progressionMethod = "isomap",
 clusterSampleSize = 500,
 resultDir = getwd(),
 saveResults = TRUE,
 saveObject = TRUE,
 saveToFCS = TRUE)

You can customize the parameters for your own need, run ?cytofkit to get information of all the parameters for cytofkit. As running with GUI, once the analysis is done, the results will be saved under resultDir automatically.

## Run with Commands (Step-by-Step)

You can make use of the functions exported from cytofkit to make your analysis more flexible and fit your own need. Here we use a sample data for demo:

### Pre-processing

## Loading the FCS data:
dir <- system.file('extdata',package='cytofkit')
file <- list.files(dir ,pattern='.fcs$', full=TRUE)
paraFile <- list.files(dir, pattern='.txt$', full=TRUE)
parameters <- as.character(read.table(paraFile, header = TRUE)[,1])

## File name
file

## [1] "/Library/Frameworks/R.framework/Versions/PrivateLibrary/cytofkit/extdata/130515_C2_stim_CD19-.fcs"

## parameters
parameters

## [1] "CD57" "IFNg" "HLA-DR" "CD62L" "CD69"
## [6] "CD16" "CD8" "TNFa" "CD85j" "CD4"
## [11] "GranzymeB" "CD27" "Vd2" "CD107a" "CD3"
## [16] "CD152" "CD19" "TIM3" "CD56" "IL10"
## [21] "CD28" "CD38" "IL4" "CD127" "IL17_CD95"
## [26] "CD95" "IL2" "GDTCR" "CCR7" "CD25"
## [31] "CD161" "CD154" "GM-CSF" "Perforin" "CD45RA"
## [36] "MIP1B"

## Extract the expression matrix with transformation
data_transformed <- cytof_exprsExtract(fcsFile = file, markers = parameters,
 comp = FALSE,
 transformMethod = "cytofAsinh")
## If analysing flow cytoetry data, you can set comp to TRUE or
## provide a transformation matrix to apply compensation

## If you have multiple FCS files, expression can be extracted and combined
combined_data_transformed <- cytof_exprsMerge(fcsFiles = file, comp=FALSE,
 markers = parameters,
 transformMethod = "cytofAsinh",
 mergeMethod = "all")
## change mergeMethod to apply different combination strategy

## Take a look at the extracted expression matrix
head(data_transformed[ ,1:3])

## (In115)Di<CD57> (Pr141)Di<IFNg> (Nd142)Di<HLA-DR>
## 130515_C2_stim_CD19-_1 0.001251318 0.003142288 0.0467847841
## 130515_C2_stim_CD19-_2 -0.001070671 0.666514724 -0.0014215565
## 130515_C2_stim_CD19-_3 0.001367652 0.001594691 0.4398952897
## 130515_C2_stim_CD19-_4 -0.002606068 1.082579674 0.4826013864
## 130515_C2_stim_CD19-_5 0.003360532 1.070186169 -0.0001361031
## 130515_C2_stim_CD19-_6 -0.001035909 0.082055890 0.0028078052

### Cell Subset Detection

## use clustering algorithm to detect cell subsets
## to speed up our test here, we only use 1000 cells
data_transformed_1k <- data_transformed[1:1000, ]

## run PhenoGraph
cluster_PhenoGraph <- cytof_cluster(xdata = data_transformed_1k, method = "Rphenograph")

## Runing PhenoGraph... Finding nearest neighbors...DONE ~ 0.172 s
## Compute jaccard coefficient between nearest-neighbor sets...DONE ~ 0.476 s
## Build undirected graph from the weighted links...DONE ~ 0.103 s
## Run louvain clustering on the graph ...DONE ~ 0.051 s
## Return a community class
## -Modularity value: 0.8094957
## -Number of clusters: 9 DONE!

## run ClusterX
data_transformed_1k_tsne <- cytof_dimReduction(data=data_transformed_1k, method = "tsne")

## Runing t-SNE...with seed 42 DONE

cluster_ClusterX <- cytof_cluster(ydata = data_transformed_1k_tsne, method="ClusterX")

## Runing ClusterX... Calculate cutoff distance...2.23
## Calculate local Density...DONE!
## Detect nearest neighbour with higher density...DONE!
## Peak detection...DONE!
## Cluster assigning...DONE!
## DONE!

## run DensVM (takes long time, we skip here)
cluster_DensVM <- cytof_cluster(xdata = data_transformed_1k,
 ydata = data_transformed_1k_tsne, method = "DensVM")

## run FlowSOM with cluster number 15
cluster_FlowSOM <- cytof_cluster(xdata = data_transformed_1k, method = "FlowSOM", FlowSOM_k = 12)

## Runing FlowSOM... Building SOM...
## Meta clustering to 12 clusters...

## DONE!

## combine data
data_1k_all <- cbind(data_transformed_1k, data_transformed_1k_tsne,
 PhenoGraph = cluster_PhenoGraph, ClusterX=cluster_ClusterX,
 FlowSOM=cluster_FlowSOM)
data_1k_all <- as.data.frame(data_1k_all)

### Cell Subset Visualization and Interpretation

## PhenoGraph plot on tsne
cytof_clusterPlot(data=data_1k_all, xlab="tsne_1", ylab="tsne_2",
 cluster="PhenoGraph", sampleLabel = FALSE)


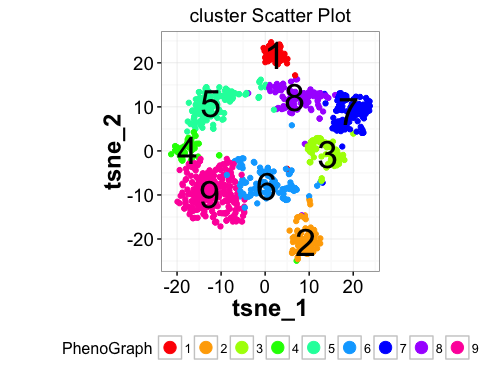


## PhenoGraph cluster heatmap
PhenoGraph_cluster_median <- aggregate(. ~ PhenoGraph, data = data_1k_all, median)
cytof_heatmap(PhenoGraph_cluster_median[, 2:37], baseName = "PhenoGraph Cluster Median")


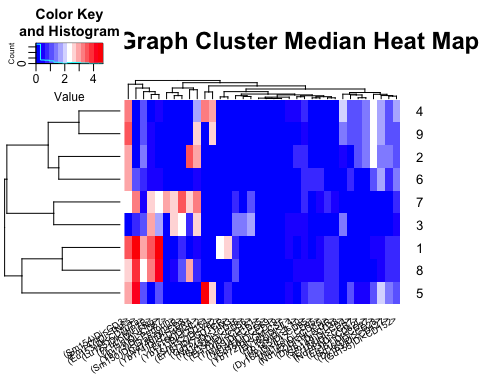


## ClusterX plot on tsne
cytof_clusterPlot(data=data_1k_all, xlab="tsne_1", ylab="tsne_2", cluster="ClusterX", sampleLabel = FALSE)


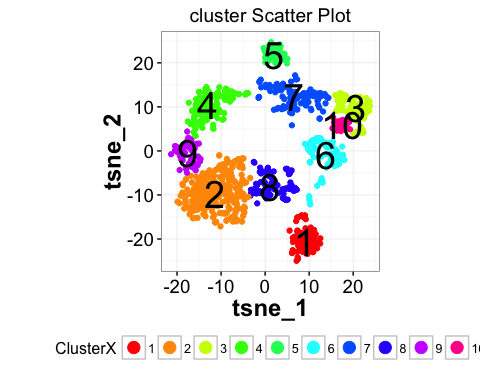


## ClusterX cluster heatmap
ClusterX_cluster_median <- aggregate(. ~ ClusterX, data = data_1k_all, median)
cytof_heatmap(ClusterX_cluster_median[, 2:37], baseName = "ClusterX Cluster Median")


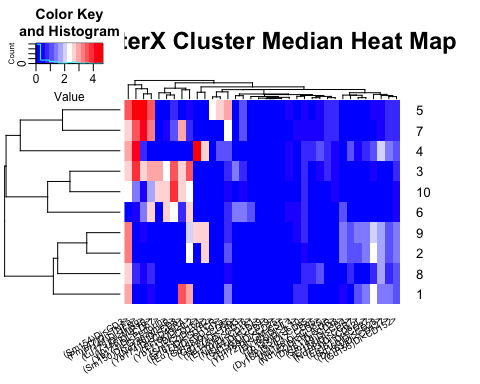


## FlowSOM plot on tsne
cytof_clusterPlot(data=data_1k_all, xlab="tsne_1", ylab="tsne_2",
 cluster="FlowSOM", sampleLabel = FALSE)


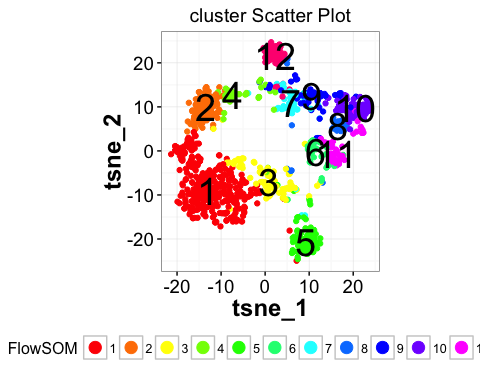


## FlowSOM cluster heatmap
FlowSOM_cluster_median <- aggregate(. ~ FlowSOM, data = data_1k_all, median)
cytof_heatmap(FlowSOM_cluster_median[, 2:37], baseName = "FlowSOM Cluster Median")


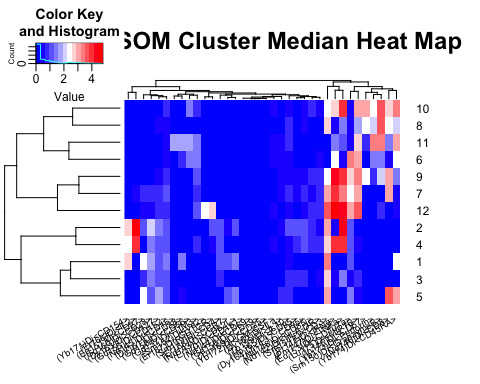


### Inference of Subset Progression

## Inference of PhenoGraph cluster relatedness
PhenoGraph_progression <- cytof_progression(data = data_transformed_1k,
 cluster = cluster_PhenoGraph,
 method="isomap", clusterSampleSize = 50,
 sampleSeed = 5)

## Runing ISOMAP... DONE

p_d <- data.frame(PhenoGraph_progression$sampleData,
 PhenoGraph_progression$progressionData,
 cluster = PhenoGraph_progression$sampleCluster,
 check.names = FALSE)

## cluster relatedness plot
cytof_clusterPlot(data=p_d, xlab="isomap_1", ylab="isomap_2",
 cluster="cluster", sampleLabel = FALSE)


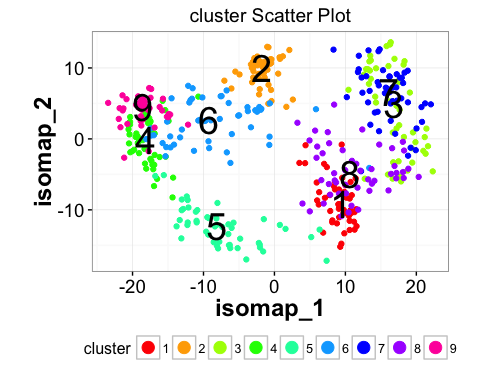


## marker expression profile
markers <- c("(Sm150)Di<GranzymeB>", "(Yb173)Di<Perforin>")

cytof_colorPlot(data=p_d, xlab="isomap_1", ylab="isomap_2", zlab = markers[1])


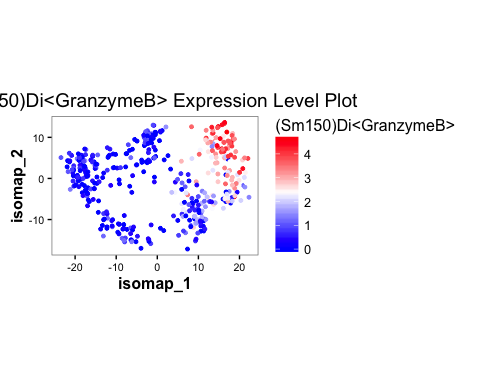


cytof_colorPlot(data=p_d, xlab="isomap_1", ylab="isomap_2", zlab = markers[2])


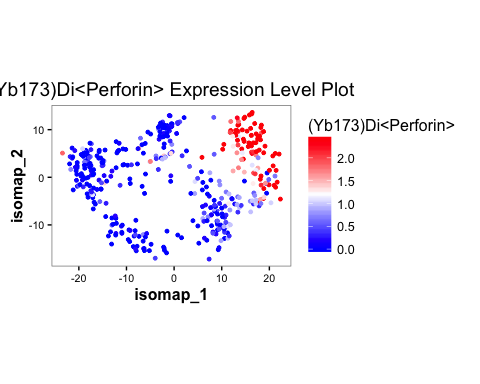


cytof_progressionPlot(data=p_d, markers=markers, orderCol="isomap_1", clusterCol = "cluster")


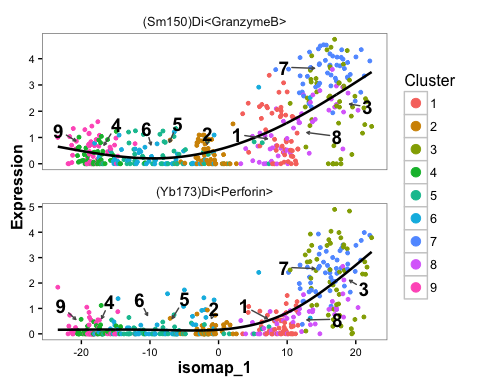


## Inference of ClusterX cluster relatedness
ClusterX_progression <- cytof_progression(data = data_transformed_1k,
 cluster = cluster_ClusterX,
 method="isomap",
 clusterSampleSize = 30,
 sampleSeed = 3)

## Runing ISOMAP... DONE

c_d <- data.frame(ClusterX_progression$sampleData,
 ClusterX_progression$progressionData,
 cluster=ClusterX_progression$sampleCluster,
 check.names = FALSE)

## cluster relatedness plot
cytof_clusterPlot(data=c_d, xlab="isomap_1", ylab="isomap_2",
 cluster="cluster", sampleLabel = FALSE)


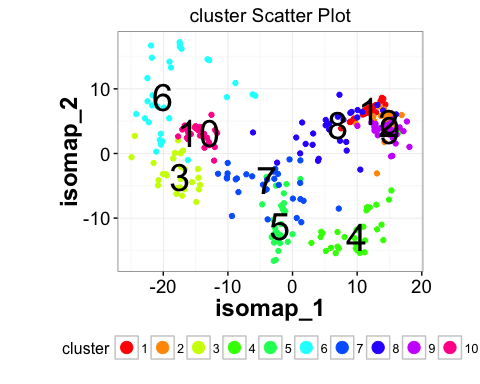


## marker expression profile
markers <- c("(Sm150)Di<GranzymeB>", "(Yb173)Di<Perforin>")

cytof_colorPlot(data=c_d, xlab="isomap_1", ylab="isomap_2", zlab = markers[1])


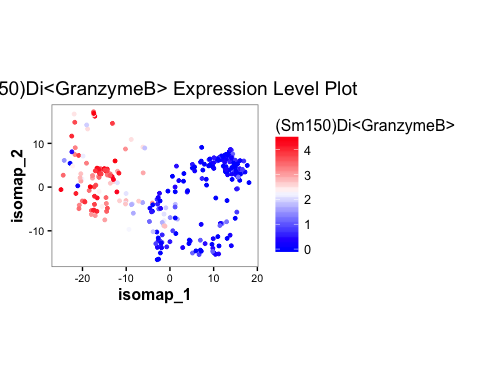


cytof_colorPlot(data=c_d, xlab="isomap_1", ylab="isomap_2", zlab = markers[2])


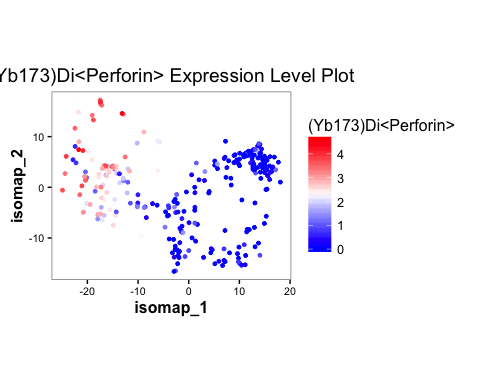


cytof_progressionPlot(data=c_d, markers, orderCol="isomap_1", clusterCol = "cluster")


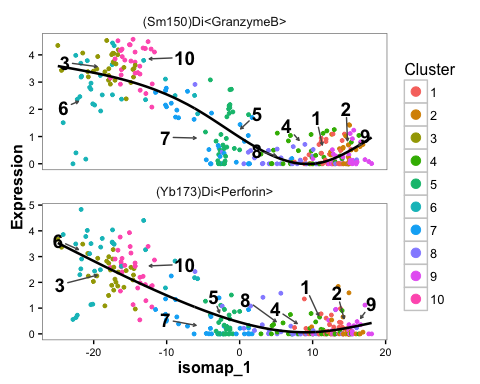


### Post-processing

## save analysis results to FCS file
cytof_addToFCS(data_1k_all, rawFCSdir=dir, analyzedFCSdir="analysed_FCS",
 transformed_cols = c("tsne_1", "tsne_2"),
 cluster_cols = c("PhenoGraph", "ClusterX", "FlowSOM"))

# Session Information

sessionInfo()

## R version 3.3.0 (2016-05-03)
## Platform: x86_64-apple-darwin13.4.0 (64-bit)
## Running under: OS X 10.11.5 (El Capitan)
##
## locale:
## [1] en_US.UTF-8/en_US.UTF-8/en_US.UTF-8/C/en_US.UTF-8/en_US.UTF-8
##
## attached base packages:
## [1] stats graphics grDevices utils datasets methods base
##
## other attached packages:
## [1] cytofkit_1.4.8 plyr_1.8.4 ggplot2_2.1.0
##
## loaded via a namespace (and not attached):
## [1] nlme_3.1-128 tsne_0.1-2
## [3] bitops_1.0-6 matrixStats_0.50.2
## [5] destiny_1.2.1 pbkrtest_0.4-6
## [7] doParallel_1.0.10 RColorBrewer_1.1-2
## [9] tools_3.3.0 vegan_2.4-0
## [11] R6_2.1.2 KernSmooth_2.23-15
## [13] rpart_4.1-10 Hmisc_3.17-4
## [15] BiocGenerics_0.18.0 mgcv_1.8-12
## [17] colorspace_1.2-6 permute_0.9-0
## [19] nnet_7.3-12 sp_1.2-3
## [21] gridExtra_2.2.1 chron_2.3-47
## [23] graph_1.50.0 quantreg_5.26
## [25] Biobase_2.32.0 formatR_1.4
## [27] SparseM_1.7 labeling_0.3
## [29] caTools_1.17.1 scales_0.4.0
## [31] DEoptimR_1.0-5 lmtest_0.9-34
## [33] mvtnorm_1.0-5 robustbase_0.92-6
## [35] proxy_0.4-16 stringr_1.0.0
## [37] digest_0.6.9 foreign_0.8-66
## [39] minqa_1.2.4 rmarkdown_0.9.6
## [41] rrcov_1.3-11 htmltools_0.3.5
## [43] lme4_1.1-12 pdist_1.2
## [45] flowCore_1.38.2 VGAM_1.0-2
## [47] FNN_1.1 shiny_0.13.2
## [49] zoo_1.7-13 gtools_3.5.0
## [51] acepack_1.3-3.3 car_2.1-2
## [53] magrittr_1.5 Formula_1.2-1
## [55] Matrix_1.2-6 Rcpp_0.12.5
## [57] munsell_0.4.3 scatterplot3d_0.3-37
## [59] stringi_1.1.1 yaml_2.1.13
## [61] MASS_7.3-45 gplots_3.0.1
## [63] Rtsne_0.11 grid_3.3.0
## [65] gdata_2.17.0 parallel_3.3.0
## [67] ggrepel_0.5 lattice_0.20-33
## [69] splines_3.3.0 knitr_1.13
## [71] tcltk_3.3.0 igraph_1.0.1
## [73] RUnit_0.4.31 boot_1.3-18
## [75] corpcor_1.6.8 reshape2_1.4.1
## [77] codetools_0.2-14 stats4_3.3.0
## [79] XML_3.98-1.4 evaluate_0.9
## [81] latticeExtra_0.6-28 data.table_1.9.6
## [83] laeken_0.4.6 vcd_1.4-1
## [85] nloptr_1.0.4 httpuv_1.3.3
## [87] foreach_1.4.3 MatrixModels_0.4-1
## [89] VIM_4.5.0 gtable_0.2.0
## [91] RANN_2.5 mime_0.4
## [93] xtable_1.8-2 RcppEigen_0.3.2.8.1
## [95] ConsensusClusterPlus_1.36.0 e1071_1.6-7
## [97] flowUtils_1.36.0 class_7.3-14
## [99] survival_2.39-5 pcaPP_1.9-60
## [101] iterators_1.0.8 FlowSOM_1.4.0
## [103] cluster_2.0.4
