# Supplementary material for: Cytofkit: A Bioconductor Package for an Integrated Mass Cytometry Data Analysis Pipeline
Source: PLoS Comput Biol. 2016 Sep 23;12(9):e1005112. doi: 10.1371/journal.pcbi.1005112 (PMC5035035; doi:10.1371/journal.pcbi.1005112)
Supplement: S3 File — (DOCX) [file pcbi.1005112.s004.docx]

cytofkit: Usage of Shiny APP

Table of Contents

Launch the Shiny APP 1

Loading .RData to Shiny APP 1

Scatter Plot 2

Run FlowSOM 3

Heat Map 4

Progression Plot 5

Subset Relationship 5

Marker Expression Profile 6

Run Diffusionmap 7

## Launch the Shiny APP

- cytofkit Shiny APP is integrated into the cytofkit pacakge, and can be deployed locally using the following command:
- require(cytofkit)
  cytofkitShinyAPP()
- cytofkit Shiny APP is also hosted by shinyapps.io, and can be used through the public link: [cytofkitShinyAPP](https://chenhao.shinyapps.io/cytofkitShinyAPP/)

## Loading .RData to Shiny APP

After the analysis was done using cytofkit, a special **RData file** will be saved with suffix of .RData. This **RData file** is saved for loading to the shiny APP to explore the results (The RData file used in this vignette can be found on the [github](https://github.com/JinmiaoChenLab/cytofkit_analysis_data_code) ).


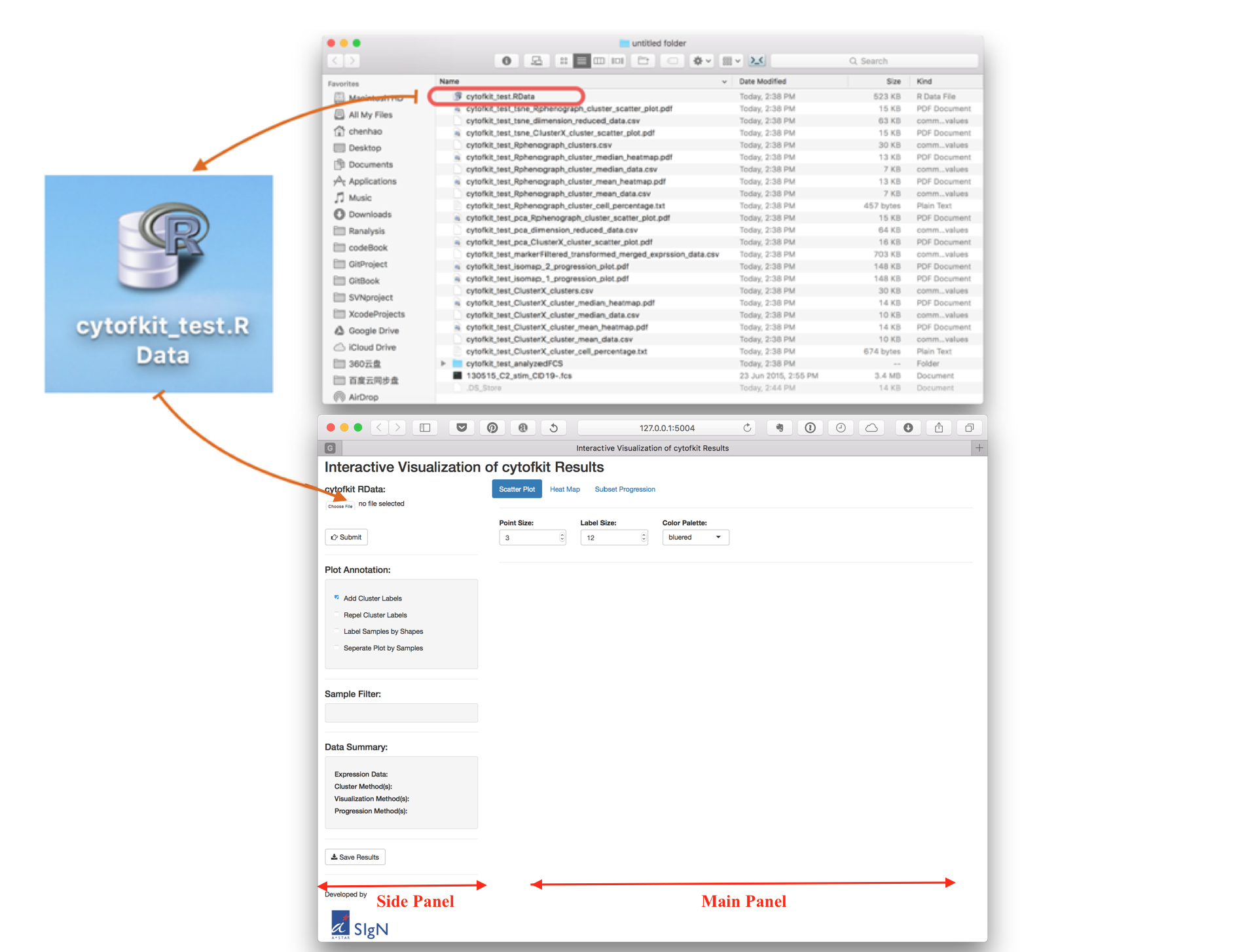


Click the up-left Choose File button to choose **RData file**, then click the submit button below to load the data.

As shown in the figure above, there are one side panel and one main panel for the cytofkit shiny APP. On the side panel, there are some global control widgets like loading the data, filtering the samples, annotating the plot and saving the results. And a summary of the data information.

## Scatter Plot

On the top of the main panel, there are three tap panels, they are **Scatter Plot**, **Heat Map** and **Subset Progression**. By default, the **Scatter Plot** will be on once the **RData file** is loaded. On the side panel, there are some control


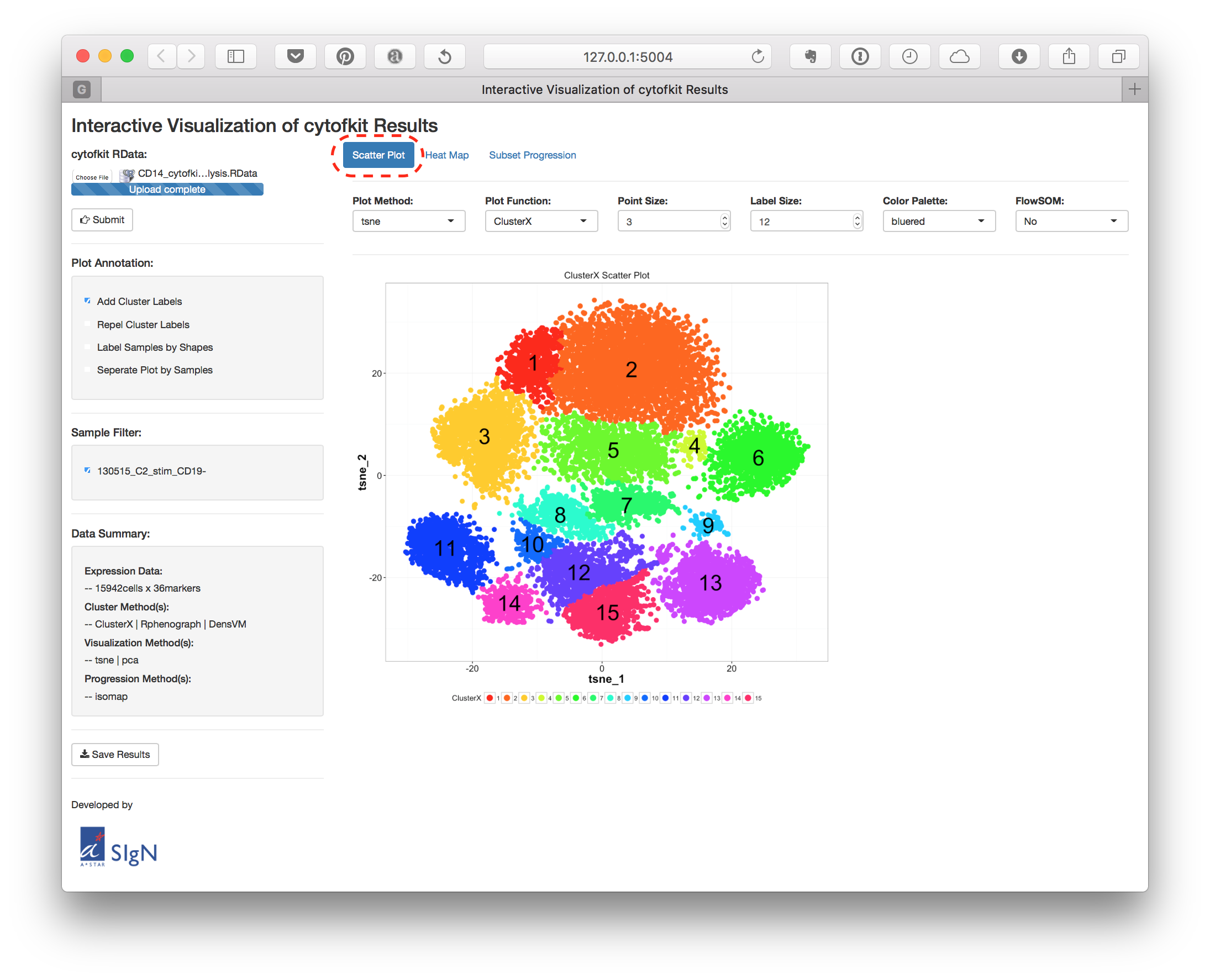


On the **Scatter Plot** main panel, you can:

- Choose the *Plot Method*, which is your data visualization methods, like t-sne, ISOMAP or PCA.
- Choose the *Plot Function*, to check the clustering results, the marker expression pattern on the scatter plot.
- Adjust the size of the points using *Point Size*.
- Adjust the cluster label size using *Label Size*.
- If you are checking the expression pattern of markers, you can change the color palette using *Color Palette*.

### Run FlowSOM

You can directly run FlowSOM clustering using the shiny APP. By selecting the *FlowSOM* to Yes, the FlowSOM clustering setup page will be shown as below:


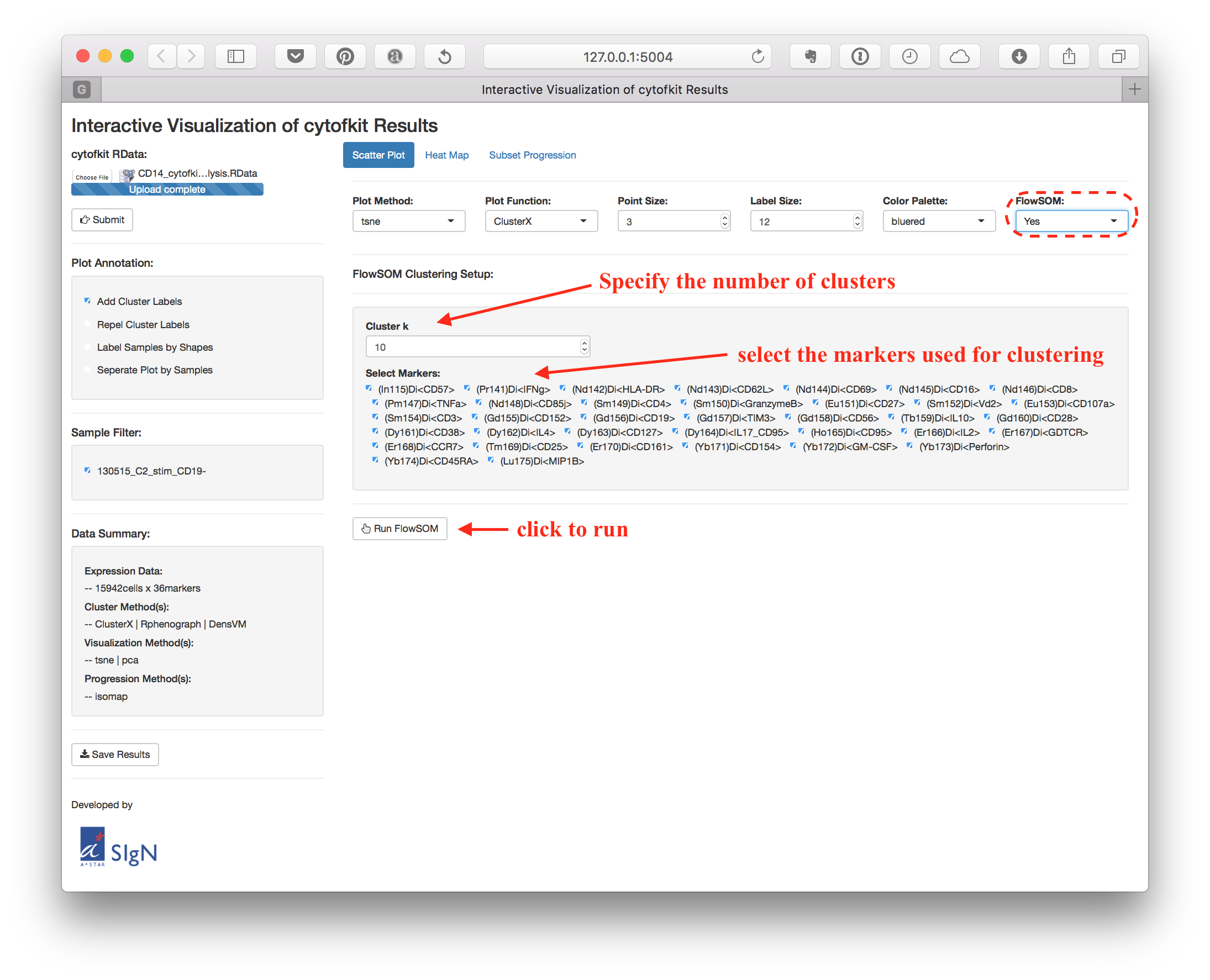


After the FlowSOM was done, the results will be added to the **RData** object and can be explored on the shiny APP.

## Heat Map

The second tap panel is the **Heat Map** panel. The mean/median expression level of markers for each cluster as well as the percentage of cells in each cluster for each sample can be visualized on this **Heat Map** panel.


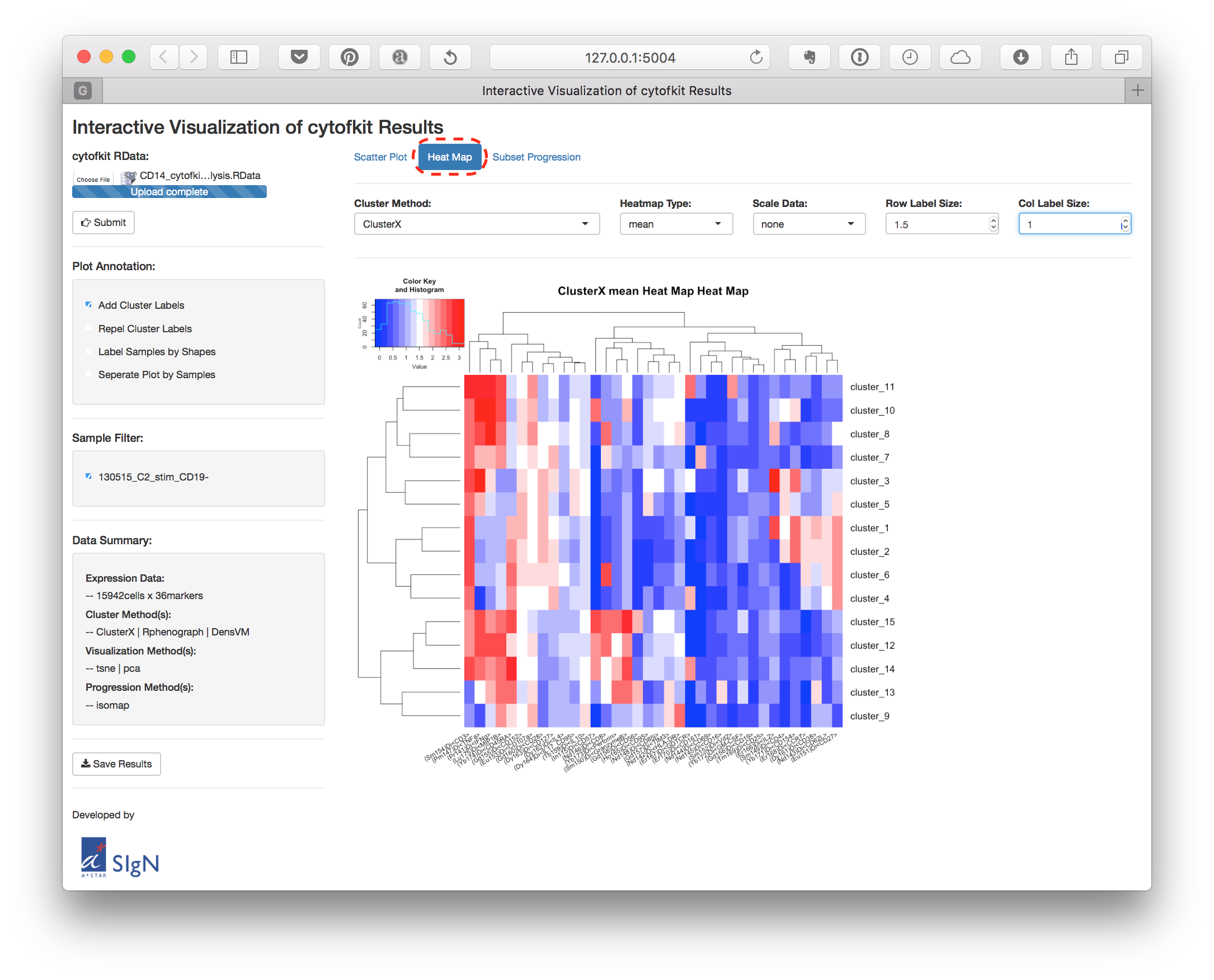


You can:

- Choose the *Cluster Method* to check the result of a selected clustering method.
- Select the *Heatmap Type* to visualize the cluster mean, median or percentage.
- Choose the *Scale Data* to scale the values by row, column or none (by default).
- Customize the size of the labels by adjusting *Row Label Size* and *Col Label Size*.

## Progression Plot

On the third tap panel, **Subset Progression** panel, there are three radio buttons which represent the *Subset Relationship*, *Marker Expression Profile* and the *Run Diffusionmap*.

### Subset Relationship

Select the *Subset Relationship*, a scatter plot showing the estimated relationship of the clusters will be displayed.


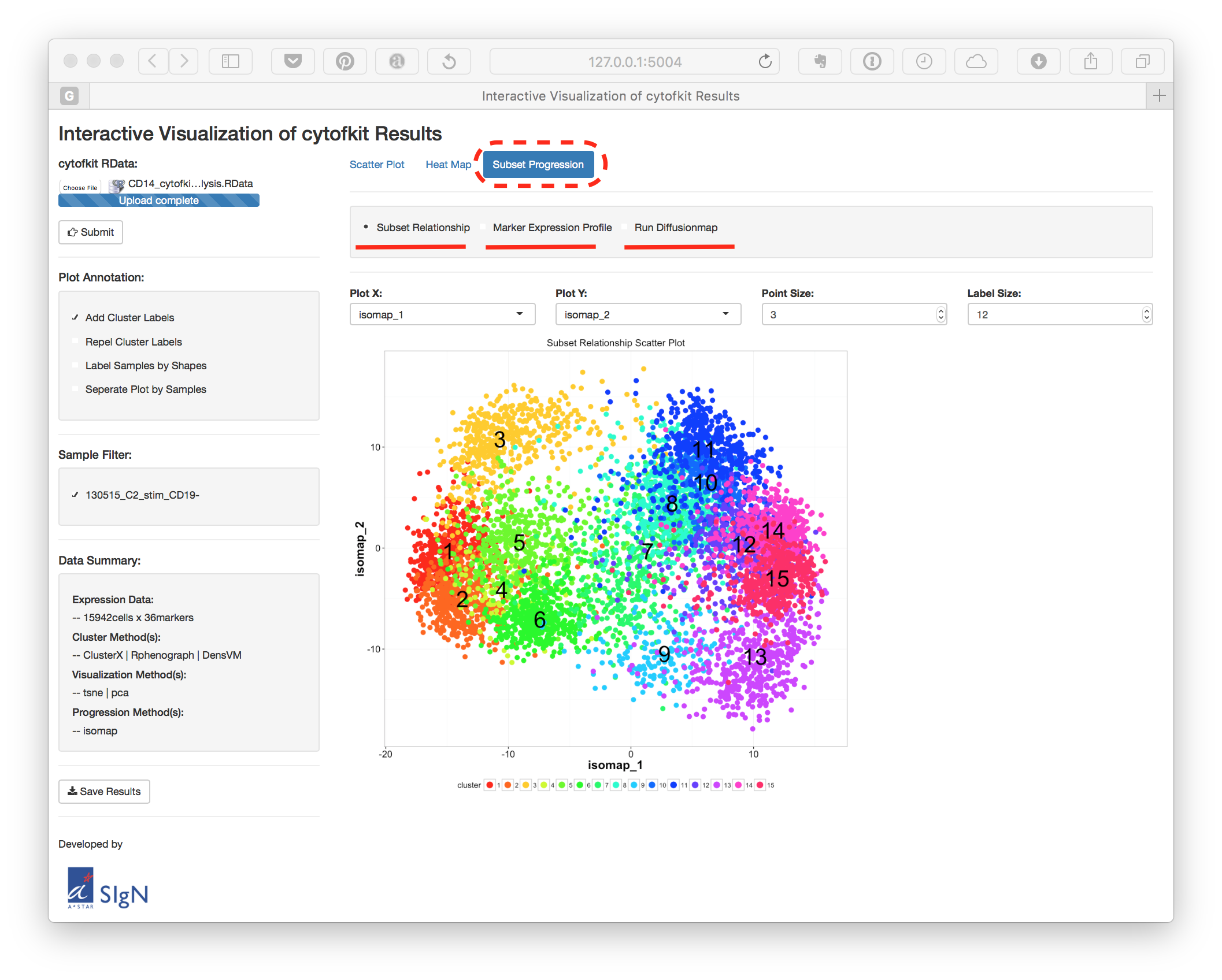


### Marker Expression Profile

Select the *Marker Expression Profile*, the selected expression trend of selected markers along a selected ordering method will be shown.


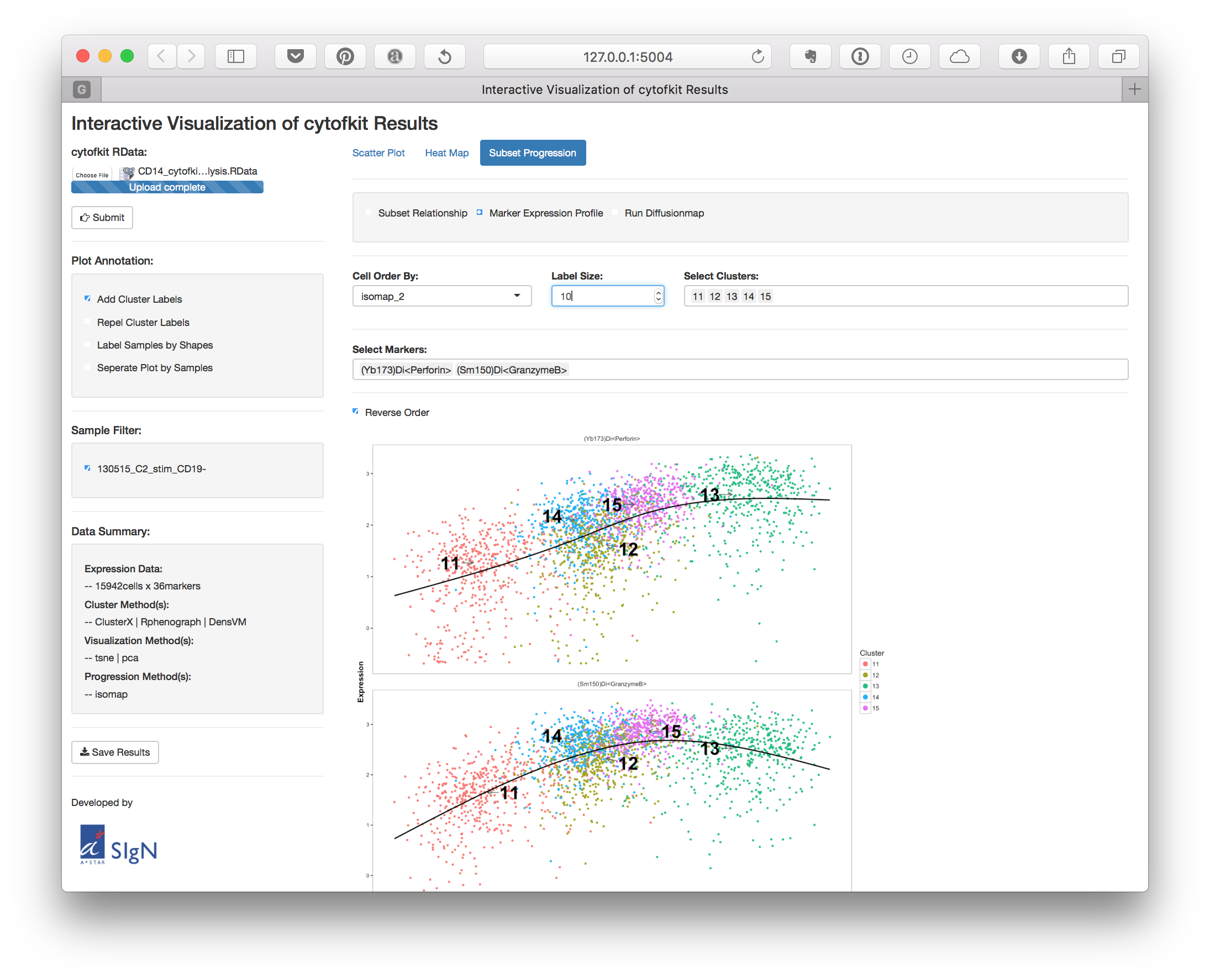


Here you can choose the *Cell Order By* method, *Select Clusters* and *Select markers* to visualize. The clusters will be color-coded, and the cluster label can be added with customized size.

### Run Diffusionmap

You can directly run Diffusion map on the shiny APP, A setup page for runing the Diffusion map will be shown when you choose *Run Diffusionmap*.


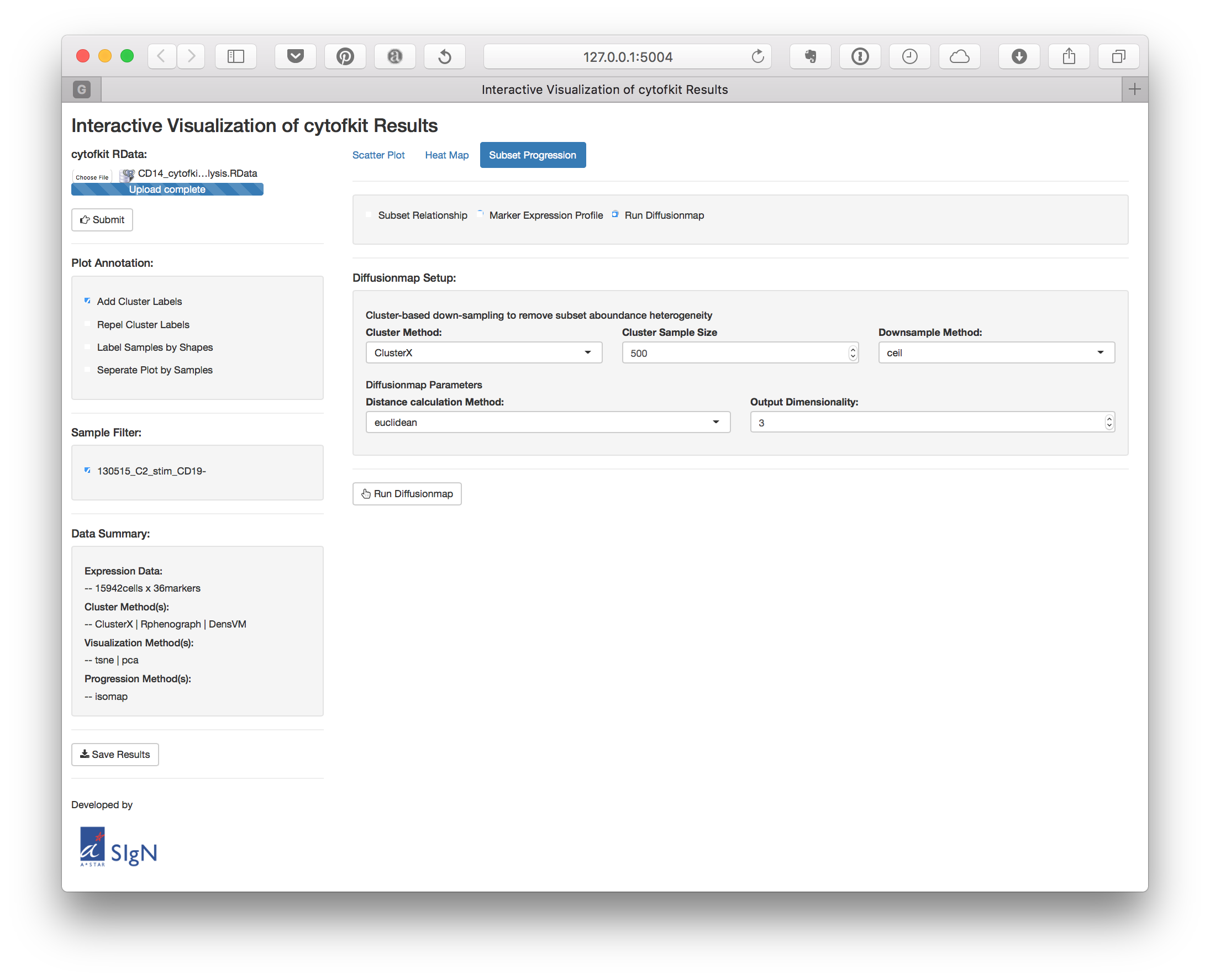


Here we combine the diffusion map with the clustering results to infer inter-subset relatedness, which is expected to help detection of cell differentiation trajectories. Firstly, we down-sampled the number of cells in each cluster to an equal size, thus reducing the cell subsets density heterogeneities and removing the dominance effect of large populations in the data. Then we ran diffusion map on the down-sampled dataset and overlay the clusters onto the transformed dimensions.

On the setup page:

- Choose a clustering results from *Cluster Method*.
- Set the down-sampling size from *Cluster Sample Size*.
- There are four different down-sampling methods, including *ceil*, *all*, *min* and *fixed*.
  - *ceil* (the default option): up to a fixed number (*Cluster Sample Size*) of cells are sampled without replacement from each cluster
  - *all*: all cells from each cluster are used.
  - *min*: The minimum number of cells among all the clusters are sampled from each cluster
  - *fixed*: a fixed num (*Cluster Sample Size*) of cells are sampled (with replacement when the total number of cell in that cluster is less than the fixe num) from each cluster.
- Select a distance calculation method for Diffusionmap.
- Select the output dimensionality for Diffusionmap.
