# Supplementary figures and images for: Cytofkit: A Bioconductor Package for an Integrated Mass Cytometry Data Analysis Pipeline
Source: PLoS Comput Biol. 2016 Sep 23;12(9):e1005112. doi: 10.1371/journal.pcbi.1005112 (PMC5035035; doi:10.1371/journal.pcbi.1005112)

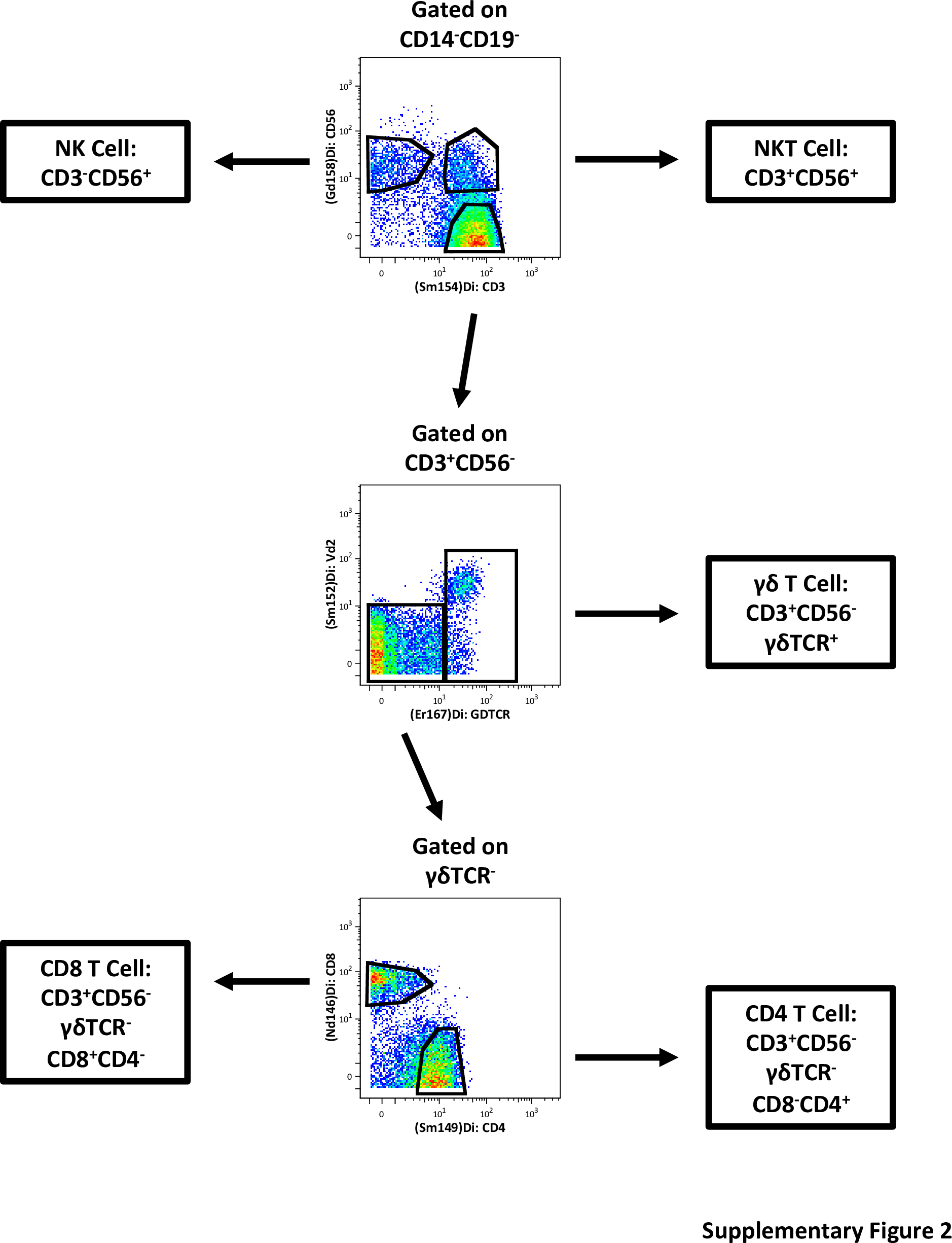

Supplement: S1 Fig — (TIF) [file pcbi.1005112.s005.tif]

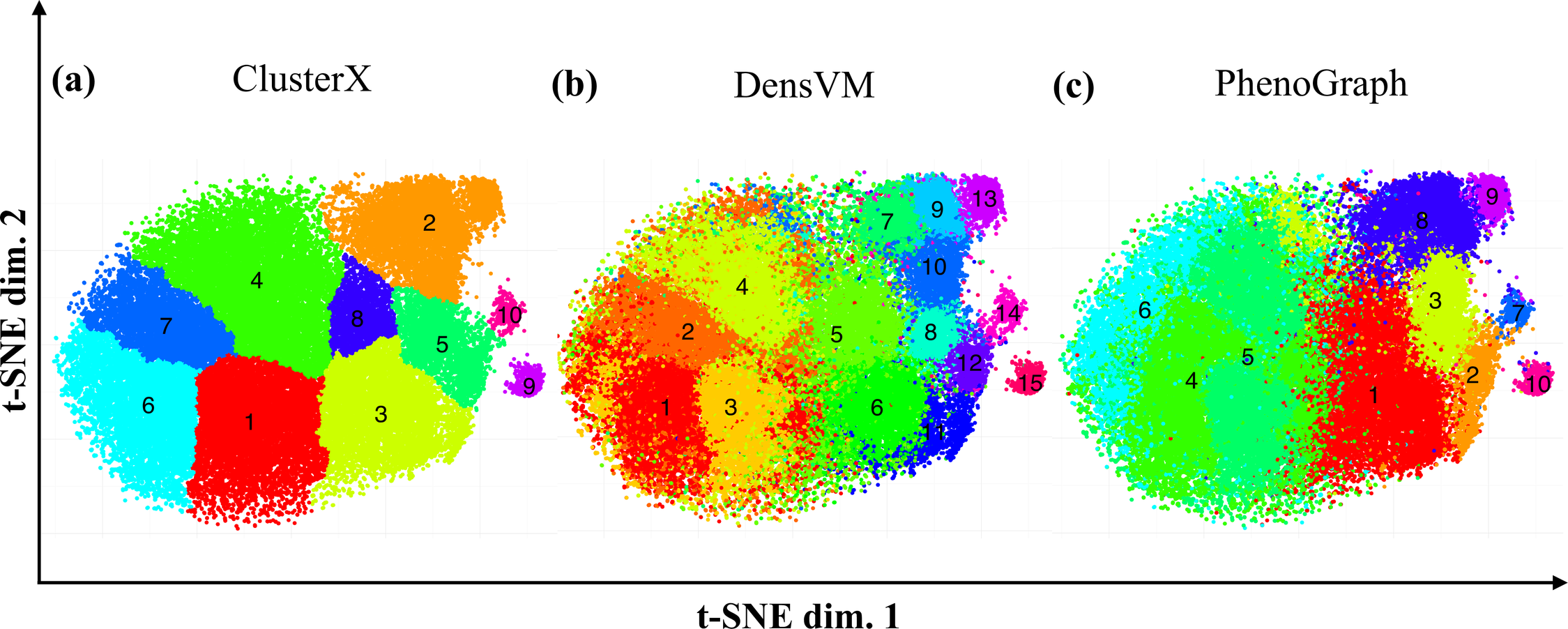

Supplement: S2 Fig — Each panel represents one clustering results mapped on the t-SNE plot; from left to right they are (a) clustering results of ClusterX, (b) clustering results of DensVM and (c) clustering results of PhenoGraph. Clusters were annotated by different colors and with cluster ID at the center of the cluster. (TIF) [file pcbi.1005112.s006.tif]

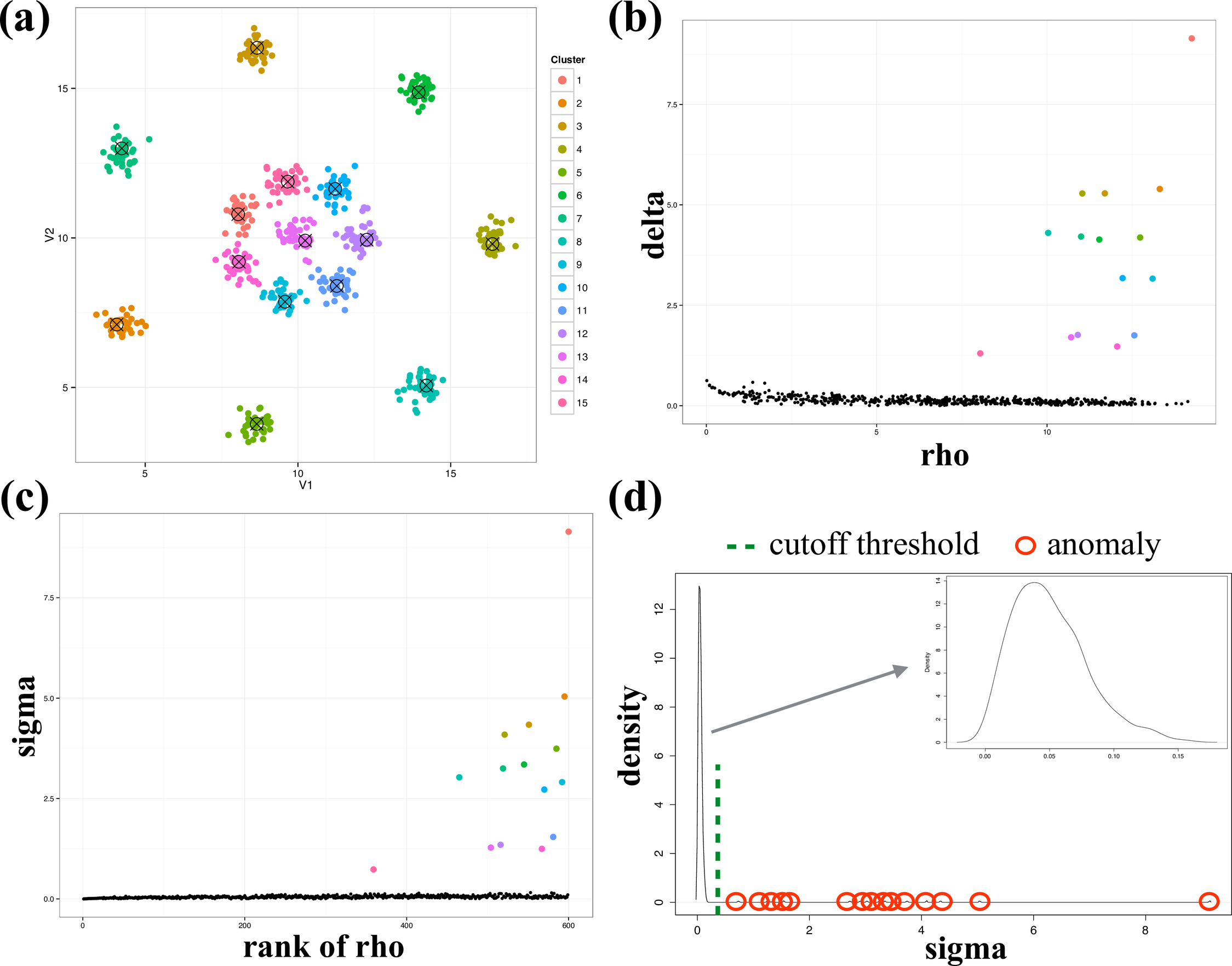

Supplement: S3 Fig — (a) Scatter plot of the D15 dataset with 15 clusters, clusters are color labeled and cluster centers are labeled by circles with crosses. (b) CFSFDP’s density peak detection method in which plots of delta against rho are generated, and users manually set a threshold point to determine the density peaks (c) ClusterX’s density peak detection method in which plots of sigma against the rank of rho are generated, and true peak points have significantly higher values of sigma. (d) ClusterX uses the generalized ESD to detect the density peaks automatically, wherein sigma is assumed to have normal distribution and peaks are regarded as anomalies that have significantly higher sigma values. (TIF) [file pcbi.1005112.s007.tif]

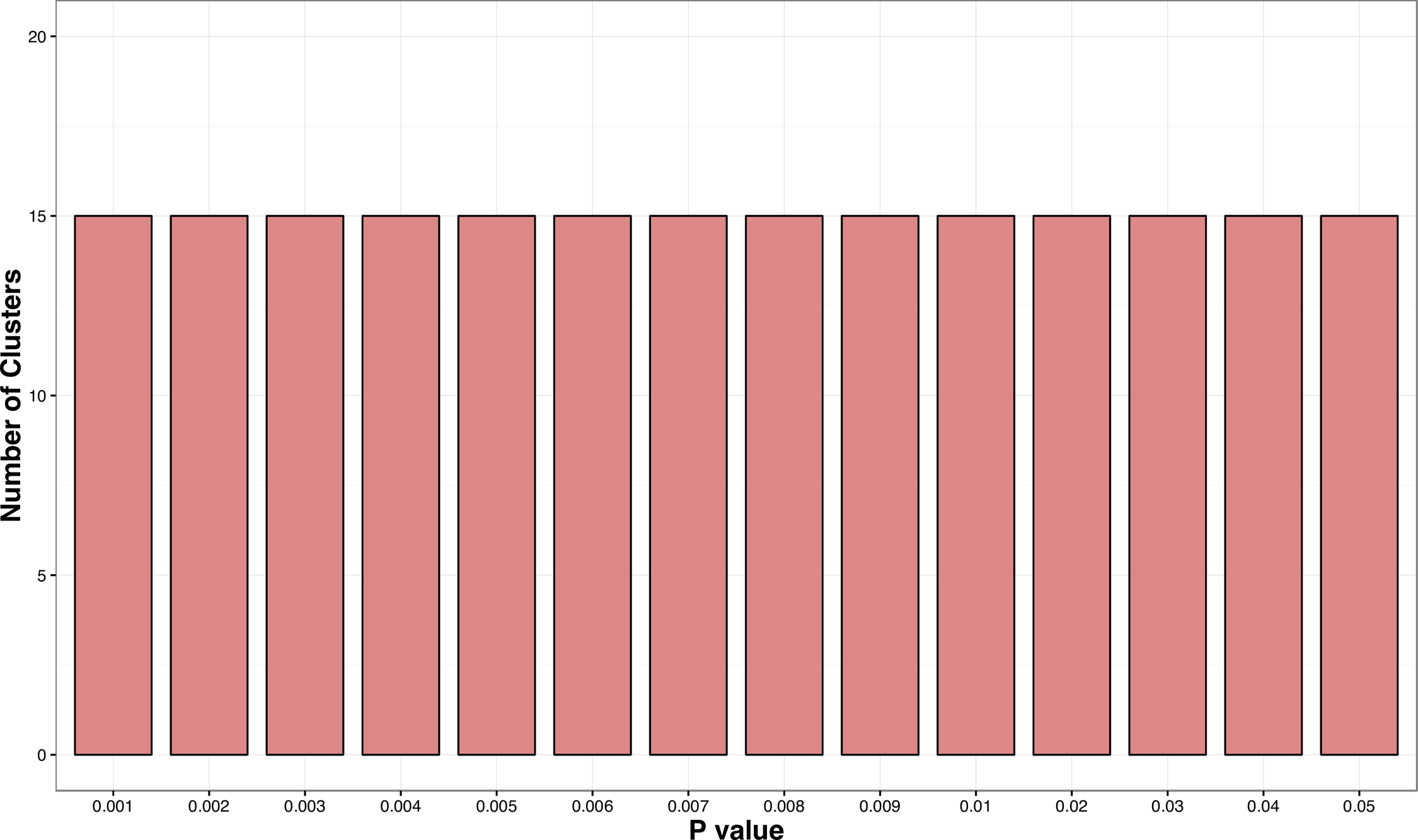

Supplement: S4 Fig — The number of density peaks is plotted over different α values within the range from 0.001 to 0.05 on the R15 datasets. (TIF) [file pcbi.1005112.s008.tif]

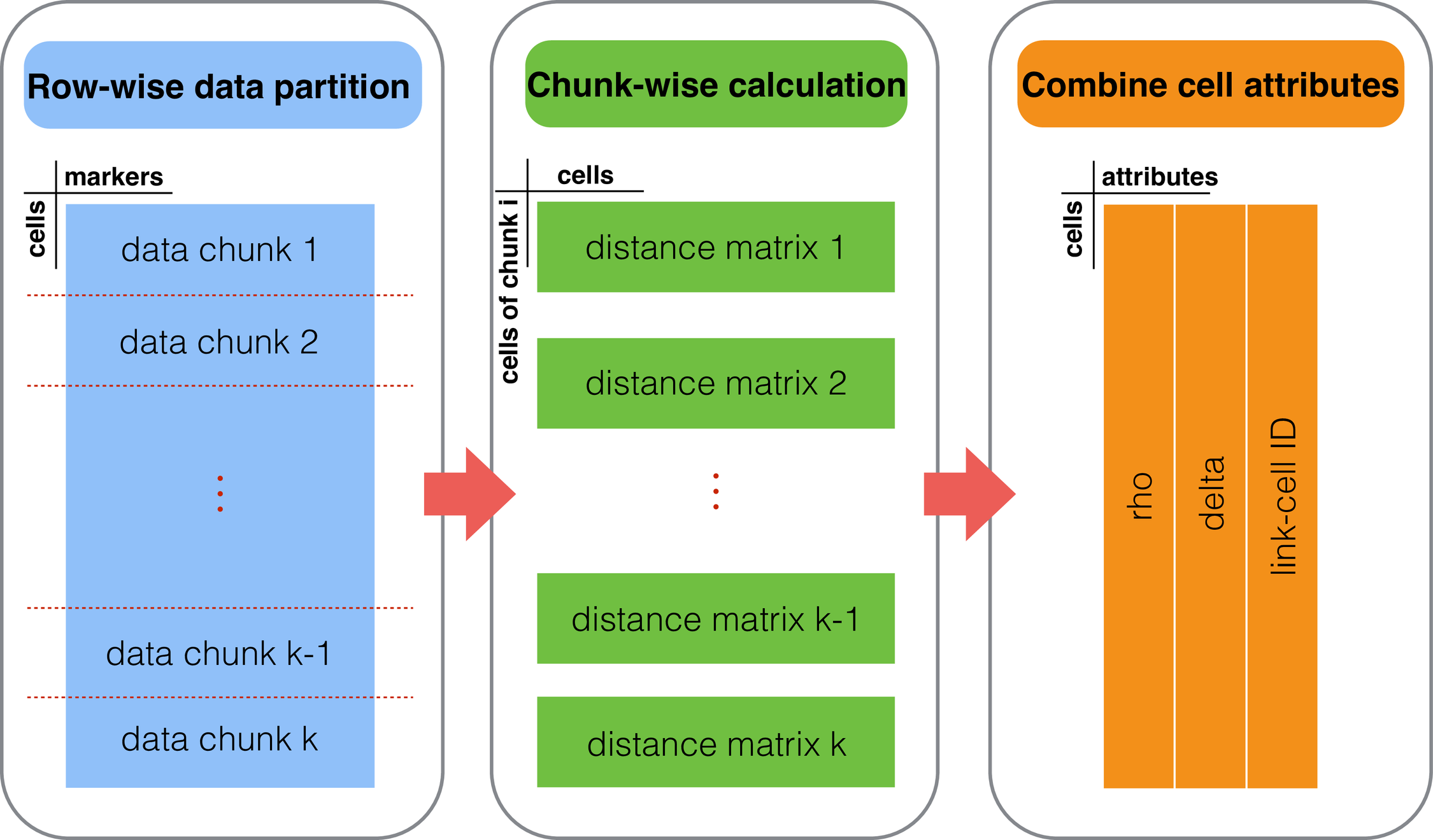

Supplement: S5 Fig — In ClusterX, data are first split row-wisely into chunks, the distance matrix is calculated in each chunk to be restricted in a limited size; then apply the calculation function for each parameters in each chunk; Finally the parameters are combined from all chunks for post processing. (TIF) [file pcbi.1005112.s009.tif]
