# Supplementary material for: Cytofkit: A Bioconductor Package for an Integrated Mass Cytometry Data Analysis Pipeline
Source: PLoS Comput Biol. 2016 Sep 23;12(9):e1005112. doi: 10.1371/journal.pcbi.1005112 (PMC5035035; doi:10.1371/journal.pcbi.1005112)
Supplement: S1 Dataset — (ZIP) [file pcbi.1005112.s001.zip › S1_dataset/cytofkit_1.4.8.tar.gz!/cytofkit/inst/doc/cytofkit_workflow.html]

cytofkit: Analysis Pipeline


# cytofkit: Analysis Pipeline

# Contents

- 1 Introduction
- 2 **cytofkit** Analysis Pipline
  - 2.1 Pre-processing
  - 2.2 Cell Subset Detection
  - 2.3 Cell Subset Visualization and Interpretation
  - 2.4 Inference of Subset Progression
  - 2.5 Post-processing
- 3 References

# 1 Introduction

The **cytofkit** package is designed to facilitate the analysis of mass cytometry data from one or multiple FCS files. User can customize their data merging strategy to combine data from multiple files with proper transformation; apply state-of-the-art clustering methods like *DensVM* [1] and *PhenoGraph* [2] as well as a in-house developed algorithm *ClusterX* to automatically detect cell subsets; visualize the high dimensional data with cluster labels by either linear transformation like *PCA* or non-linear dimensionality reduction like *ISOMAP* [3] or *t-SNE* [4]; map the progression of cellular subtypes estimated by *ISOMAP* or *Diffusion map* [5] and profile the marker expression along the cell progression. In addition to providing the research community with an one-stop analysis platform, **cytofkit** is shipped together with an intuitively designed GUI and an interactive web app to facilitate the easy analysis and interpretation of the mass cytometry data.

# 2 **cytofkit** Analysis Pipline

A schematic overview of the **cytofkit** pipeline is as below, it contains four major components:

- Pre-processing
- Cell subset detection
- Cell subset visualization and interpretation
- Inference of subset progression.

## 2.1 Pre-processing

In this step, one or multiple selected FCS files will be loaded into the R environment using the `read.FCS` function of **flowCore** package. Expression values of selected markers are then extracted and transformed with a proper transformation method (including *cytofAsinh* (suggest for CyTOF data), *autoLgcl* (suggest for FCM data), *logicle* and *arcsinh*), expression matrixes from multiple files are then combined into a single matrix. For multiple FCS files, **cytofkit** provides four data combining methods including i) *ceil* which samples up to a user specified number of cells without replacement from each FCS file then combine, ii) *all* which takes all cells from each FCS file then combine, iii) *min* which samples a minimum number of cells among all FCS files from each FCS file then combine and iv) *fixed* which samples an user specified number of cells (with replacement when the total number of cell in the file is less than the specified number) from each FCS file then combine. After combination, the whole dataset will be stored in a data matrix, with row names represented as *filename\_cellID*, column mames represented as *marker\_name*. This step is implemented in funciton `cytof_exprsMerge`, read the details of this funcion:

```
?cytof_exprsMerge
```

## 2.2 Cell Subset Detection

The subset detection is performed by clustering algorithms. **cytofkit** is shipped with two state-of-the-art clustering methods *DensVM* and *PhenoGraph* and an in-house developed clustering algorithm *ClusterX*. Among them, *DensVM* and *ClusterX* are density-based clustering algorithms estimated from the *t-SNE* embedded map. While *PhenoGraph* is a graph based clustering algorithm performed directly on the high dimensional data. For version later than 1.4.4, we plugged in the *FlowSOM* clustering algorithm [6]. Check the function `cytof_cluster` for implementation of this step.

```
?cytof_cluster
```

## 2.3 Cell Subset Visualization and Interpretation

Three dimensionality reduction methods are integrated into **cytofkit** for visualizing the high dimensional mass cytometry data into an information-condensed and relationship-preserved two-dimensional map. These included one linear transformation method *PCA* and two non-linear transformation methods *ISOMAP* and *t-SNE*. *PCA* is a widely used technique to remove the linear correlation between variables. After *PCA*, data is represented by principal components, and those two or three principal components with highest variance are used to visualize the data. *ISOMAP* is used to embed the high dimensional data points into low dimensions while preserving the intrinsic geometry of the data structure. *t-SNE* finds the two-dimensional representation of high-dimensional data that best preserve both local and global geometry. After dimensional reduction, **cytofkit** plots the cell points into these transformed two-dimensional map with point color representing its cell type detected from last step, and point shape representing which sample the cell is from. To summarize the expression profile of each cell subset, a heat map can be generated to visualize the mean or median expression for each marker in each cell type. This heat map can greatly facilitate the interpretation of known cell types with prior knowledge of their special marker expression features as well as detection of new cell types with novel expression patterns. Check the function `cytof_dimReduction` for implementation of this step.

```
?cytof_dimReduction
```

## 2.4 Inference of Subset Progression

Instead of inferring the subset progression using all cells, we perform the progression path estimation based on the detected subsets of cells. Specifically, we down sample the number of cells to a fixed number for each cell subset, thus to make every subset has the equal number of cells. This removes the potential dominant influence from cell subtypes with most number of cells. Then *Diffusion Map* or *ISOMAP* is used to infer the relationships among the down sampled subsets. *Diffusion Map* and *ISOMAP* are both able to compare the overall relatedness of cells and embed the cells taking into account the nonlinear geodesic distances between cells. By plotting the marker expression along the *Diffusion Map* or *ISOMAP* dimension 1 or dimension 2 with lowess regression, markers with expression patterns that highly correlated with progression can be defined as regulatory markers. Check the function `cytof_progression` for implementation of this step.

```
?cytof_progression
```

## 2.5 Post-processing

The cluster results can be visualized with dimension transformed data from *t-SNE*, *PCA* and *ISOMAP*. The cluster coordinates, together with the *t-SNE*, *PCA* and *ISOMAP* coordinates, will be stored to the FCS files as additional parameters and for post analysis, all result files and plots can be saved using the function `cytof_writeResults`.

```
?cytof_writeResults
```

Among the saved results, an R data object will be saved with suffix of *.RData*, this is specially for loading to the shiny web APP for interactive visualization and exploration of the analysis results. The shiny APP can be launched with command `cytofkitShinyAPP()` or through this link https://chenhao.shinyapps.io/cytofkitShinyAPP/.

# 3 References

[1] Becher B, Schlitzer A, Chen J, Mair F, Sumatoh HR, Wei K, et al. High-dimensional analysis of the murine myeloid cell system. Nat Immunol. 2014;15.doi:10.1038/ni.3006.nature

[2] Levine JH, Simonds EF, Bendall SC, Davis KL, Amir ED, Tadmor MD, et al. Data-Driven Phenotypic Dissection of AML Reveals Progenitor-like Cells that Correlate with Prognosis. Cell. Elsevier Inc.; 2015; 1???14. doi:10.1016/j.cell.2015.05.047

[3] Tenenbaum JB, de Silva V, Langford JC, Silva V De, Langford JC. A global geometric framework for nonlinear dimensionality reduction. Science. American Association for the Advancement of Science; 2000;290: 2319-23. doi:10.1126/science.290.5500.2319

[4] Maaten L Van Der. Accelerating t-SNE using Tree-Based Algorithms. J Mach Learn Res. 2014;15: 1-21.

[5] Haghverdi L, Buettner F, Theis FJ. Diffusion maps for high-dimensional single-cell analysis of differentiation data. Bioinformatics. 2015; 1-10. doi:10.1093/bioinformatics/btv325

[6] Van Gassen S, Callebaut B, Van Helden MJ, Lambrecht BN, Demeester P, Dhaene T, et al. FlowSOM: Using Self-Organizing Maps for Visualization and Interpretation of Cytometry Data. Cytometry A. 2015; 1-10. doi:10.1002/cyto.a.22625
